# Cooperative Amidase Systems Control Product Selectivity During Enzymatic Nylon Depolymerization

**DOI:** 10.1002/mabi.70274

**Published:** 2026-10-15

**Authors:** Sukmanita Dewi, Nico Bäse, Alan Wypych, Hendrik Puetz, Christoph Janknecht, Ulrich Schwaneberg, Wolfgang R. Streit, Laura Hartmann

**Affiliations:** ^1^ Institute of Macromolecular Chemistry Albert‐Ludwigs‐University of Freiburg Freiburg Germany; ^2^ Department of Microbiology and Biotechnology Biocenter Klein Flottbek University of Hamburg Hamburg Germany; ^3^ Institute of Biotechnology RWTH Aachen University Aachen Germany; ^4^ Freiburg Materials Research Center Albert‐Ludwigs‐University of Freiburg Freiburg Germany

## Abstract

Recent advances in enzymatic polyester depolymerization have highlighted the potential of biocatalysis for sustainable plastic recycling. In contrast, efficient enzymatic strategies for polyamide (PA) degradation remain limited. Here, we investigate how complementary amidases influence product formation during the enzymatic treatment of bulk nylon‐6 (PA6) and nylon‐6,6 (PA66). Guided by previously established substrate preferences, enzyme combinations are designed to couple NylC‐mediated oligomer cleavage with downstream conversion by NylB or cyclic oligomer ring‐opening by NylA. Sequential enzyme treatments demonstrate efficient conversion of characteristic PA6‐ and PA66‐derived intermediates, while one‐pot systems enable coordinated downstream conversion at 34°C–40°C. For PA6, NylB efficiently converts the NylC‐generated 6‐aminohexanoic acid dimer to 6‐aminohexanoic acid, whereas for PA66, NylA selectively redirects accessible cyclic PA66‐derived species toward the corresponding linear repeat‐unit product. These changes in product distribution are not accompanied by a substantial increase in the overall amount of soluble polymer‐derived material. The detected soluble products represent less than 1% of the initial polymer mass under the investigated conditions, demonstrating that overall soluble product release remains limited. Together, these results show that complementary amidases can control downstream intermediate conversion and product selectivity during enzymatic nylon depolymerization.

## 1 Introduction

The growing accumulation of persistent plastic waste has intensified the need for sustainable recycling strategies. Enzymatic depolymerization has emerged as a promising approach for the selective cleavage of synthetic polymers under mild reaction conditions, with particularly substantial progress achieved for polyesters such as polyethylene terephthalate (PET). Engineered PET hydrolases have demonstrated markedly improved polymer hydrolysis and illustrate the potential of biocatalytic approaches for polymer recycling [1, 2, 3, 4]. In addition to enzyme engineering, combinations of catalysts with complementary substrate preferences can facilitate downstream intermediate conversion and thereby influence overall product distributions. Such multienzyme strategies are well established for the degradation of natural polymers and have increasingly also been investigated for synthetic polymer systems [5, 6, 7, 8, 9]. In PET recycling, complementary enzymes have been used to convert soluble intermediates generated during polymer hydrolysis [8, 10, 11, 12]. Multienzyme approaches have additionally been explored for mixed polyester–amide materials, as demonstrated by de Witt and co‐workers using leaf‐branch‐compost cutinase (LCC) and a NylC amidase for poly(ester amide) degradation [13].

Inspired by these advances, attention has increasingly turned toward extending enzymatic depolymerization strategies to polyamides (PAs), another major class of engineering polymers [12]. Among these materials, nylon‐6 (PA6) and nylon‐6,6 (PA66) represent the most industrially relevant PAs with applications in textiles, engineering materials, packaging, and automotive components [12, 14, 15]. However, translating enzymatic recycling concepts from polyesters to PAs has proven considerably more challenging. Strong intermolecular hydrogen bonding between amide groups promotes dense chain packing and high crystallinity in nylon materials, which severely restricts enzymatic accessibility to amide bonds [16, 17]. Consequently, reported degradation yields for nylon materials remain in the low single‐digit percentage range [18, 19, 20].

Despite these challenges, several microorganisms capable of utilizing nylon‐derived oligomers as carbon sources have been identified, leading to the discovery of a small group of nylon‐active hydrolases [21, 22, 23]. Among these, the enzymes NylA, NylB, and NylC from *Paenarthrobacter ureafaciens*, previously described as *Arthrobacter* sp. KI72, have been studied most extensively and serve as model systems for nylon degradation [13, 24, 25, 26, 27]. These enzymes exhibit distinct catalytic functions for cleaving synthetic PA substrates. NylC preferentially cleaves internal amide bonds within nylon oligomers, generating shorter oligomeric products, while NylB preferentially hydrolyzes terminal amide bonds in shorter oligomeric substrates. In contrast, NylA hydrolyzes cyclic nylon oligomers through ring‐opening reactions [24, 25]. Together, these enzymes constitute a functionally complementary nylon‐oligomer degradation system whose catalytic roles have been established through extensive biochemical and structural studies [24].

Although the catalytic functions of these nylon hydrolases are well established, the consequences of deliberately combining their complementary activities during treatment of heterogeneous bulk PA substrates remain less clearly resolved. In such systems, polymer accessibility, release of soluble oligomers, and subsequent enzymatic conversion can occur simultaneously, complicating mechanistic interpretation. To resolve individual cleavage events under defined conditions, we previously employed sequence‐defined nylon oligomers as analytically tractable model substrates to characterize cleavage preferences of the investigated amidases [20]. Previous studies have additionally demonstrated complementary NylC/NylB activity during PA6 hydrolysis [26, 28]. Building on this established framework, the present study investigates how complementary amidases influence downstream intermediate conversion and product selectivity when applied sequentially or simultaneously to bulk PA6 and PA66 substrates.

The experimental concept is summarized in Figure 1. Guided by established substrate preferences, NylC variants were combined with NylB for PA6 and with NylA for PA66 [24, 27, 29]. These combinations were evaluated first in sequential enzyme treatments, allowing characteristic soluble intermediates to accumulate before addition of the complementary enzyme, and subsequently in temperature‐compatible one‐pot systems. This design allowed us to assess how selective downstream conversion alters soluble product distributions and whether these changes are accompanied by increased overall release of soluble polymer‐derived products.

**FIGURE 1:**
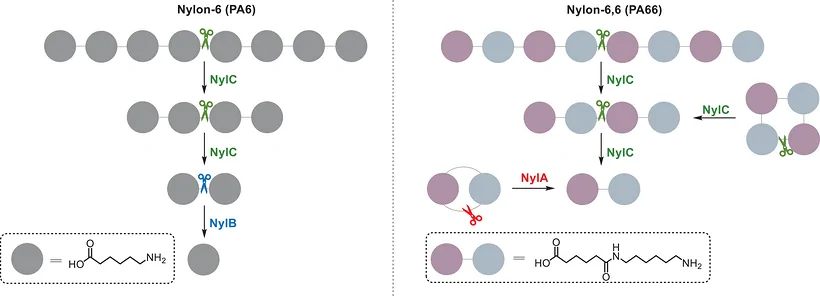
Conceptual model of enzymatic depolymerization pathways for nylon‐6 and nylon‐6,6.

## 2 Experimental Section

### 2.1 Materials

PA6 film (0.2 mm thickness) and PA66 granules (3 mm diameter) were purchased from Goodfellow GmbH (Hamburg, Germany). The crystallinity of the untreated substrates was determined by differential scanning calorimetry (DSC) as 27.7% for PA6 and 33.4% for PA66; the corresponding DSC thermograms and analysis details are provided in the Supporting Information (Figures S1 and S2). PA6 film was punched into 6.4 mm diameter discs (7.4 mg per disc) using a 12 kN 96‐well puncher (Gechter GmbH, Obermichelbach, Germany). 6‐Aminohexanoic acid (6‐AHA, 99.8%), *ε*‐caprolactam (99.8%), and adipic acid were obtained from Sigma‐Aldrich. Defined PA6 and PA66 degradation product standards, including the 6‐AHA dimer, 6‐AHA trimer, linear PA66 repeat‐unit product (lin‐M), and PA66 dimer were synthesized according to our previously reported solid‐phase synthesis protocol [20].

### 2.2 Enzyme Expression and Purification

Four amidases were used in this study: NylA, NylB, NylC_p2_‐GYAQ (hereafter referred to as NylC_p2_‐TS), and the engineered variant NylC_p2_‐TS^F134W/D304M/R330A^ (hereafter referred to as NylC‐HP). NylA, NylB, and NylC_p2_‐TS were heterologously expressed in *Escherichia coli* and purified according to established protocols [27, 30, 31, 32]. NylC‐HP was expressed and purified following the procedure reported by Puetz et al. [29]. Detailed expression and purification procedures, together with amino acid sequences of all enzyme variants, are provided in the Supporting Information.

### 2.3 Bulk Polyamide Hydrolysis Assays

Enzyme reactions were performed in 100 mM potassium phosphate buffer (KPi, pH 7.5) containing 150 mM NaCl. Reactions were conducted at enzyme‐specific temperatures (34°C for NylA [33], 40°C for NylB [33], 50°C for NylC_p2_‐TS [21, 26, 32], and 70°C for NylC‐HP [29]). PA6 assays contained one disc (Ø 6.4 mm, 7.4 mg; 0.67 wt% substrate loading) in a total reaction volume of 1.1 mL, whereas PA66 assays contained one granule (Ø 3 mm, 11 mg; 2.2 wt% substrate loading) in a total volume of 0.5 mL. Total enzyme concentrations were 1.1 µM for PA6 and 1.65 µM for PA66, corresponding to an enzyme‐to‐substrate ratio of 0.15 mM g^−1^. Based on the respective reaction volumes, these conditions correspond to substrate‐normalized total enzyme loadings of 0.164 µmol g^−^^1^ PA6 and 0.075 µmol g^−^^1^ PA66, respectively.

All reactions were incubated at 400 rpm in a Thermomixer C (Eppendorf SE, Hamburg, Germany). Aliquots were collected at defined time points every 24 h and quenched by ultrafiltration using centrifugal filters (10 kDa molecular weight cutoff, VWR International GmbH, Darmstadt, Germany) at room temperature (5 min, 13,500 rpm), thereby separating residual polymer particles and enzyme from the soluble hydrolysis products. The filtrates were subsequently analyzed by RP‐HPLC and ESI‐MS to quantify soluble hydrolysis products. Corresponding no‐enzyme controls were processed under the same analytical workflow and were used to distinguish enzyme‐dependent product formation from soluble low‐molecular‐weight species inherently present in or released from the polymer substrates (see Figures S3 and S4).

### 2.4 LC‐MS Product Quantification

Soluble hydrolysis products were analyzed by RP‐HPLC‐MS using an Agilent Technologies 6120 series system equipped with a binary pump, a C18 reversed‐phase column (3.0 × 50 mm, 3 µm particle size; MZ‐Aqua Perfect C18, Agilent Technologies, Waldbronn, Germany), a quadrupole mass spectrometer with electrospray ionization (ESI‐MS, m/z 100–2000), and a UV/Vis detector operated at 205 nm. Mobile phase A consisted of water containing 0.1% (v/v) formic acid, and mobile phase B consisted of acetonitrile. All analyses were performed with an injection volume of 5 µL at a flow rate of 0.4 mL min^−1^.

For quantification of PA6‐derived monomeric and dimeric products, chromatographic separation was carried out using 100% mobile phase A for 5 min, followed by a linear gradient from 0% to 10% mobile phase B over 15 min. Under these conditions, 6‐AHA monomer, *ε*‐caprolactam, and the 6‐AHA dimer were identified at retention times (t_R_) of 1.9, 11.8, and 14.3 min, respectively (Figure S5). For analysis of higher oligomers and PA66‐derived hydrolysis products, chromatographic separation was performed using 100% A for 3 min, followed by a linear gradient from 0% to 50% B over 15 min. Under these conditions, the 6‐AHA trimer eluted at t_R_ = 9.7 min (Figure S6), while the linear PA66 repeat‐unit product (lin‐M) and PA66 dimer eluted at t_R_ values of 7.9 and 10.6 min, respectively (Figure S8).

Product concentrations were determined using calibration curves generated from authentic standards synthesized as described above (Figures S7 and S9). Species lacking isolated standards were quantified using structurally related surrogate calibration curves based on comparable amide content and UV response. For cyclic PA66 monomeric species (cy‐Mono), the calibration curve of the corresponding linear repeat‐unit product (lin‐M) was used together with an empirical response correction factor derived from enzymatic cy‐Mono conversion experiments to account for differences in detector response between cyclic and linear species (see Supporting Information for details). Because an authentic cy‐Mono standard was not available, the resulting cy‐Mono concentrations should be regarded as semi‐quantitative estimates rather than absolute concentrations. The cyclic PA66 dimer (cy‐Dimer) and the diacid‐terminated product (M–AA; adipic acid–hexamethylenediamine–adipic acid) were quantified using the calibration curve of the corresponding PA66 dimer. Hexamethylenediamine (HMDA) was not quantified using the applied RP‐HPLC method. Accordingly, the PA66 product analysis does not represent a complete quantitative mass balance of hydrolysis to adipic acid (AA) and HMDA.

## 3 Results and Discussion

To investigate how complementary amidase systems influence soluble product formation during enzymatic treatment of bulk PAs, hydrolysis assays were first established under standardized conditions using enzyme loadings corresponding to an enzyme‐to‐substrate ratio of 0.15 mM g^−^^1^, consistent with the benchmark conditions reported by Bell et al. for PA6 hydrolysis studies [21]. This loading was maintained across all single‐enzyme, sequential, and simultaneous experiments so that differences in product formation could be attributed to enzyme cooperation rather than catalyst loading.

The present study focuses on soluble product formation and downstream intermediate conversion rather than on a complete quantitative assessment of bulk‐polymer conversion. Molecular‐weight changes of the residual polymer were not determined by SEC, and the commercial PA substrates were used without prior organic‐solvent extraction. Consequently, particularly for PA66, part of the detected cyclic product pool may originate from low‐molecular‐weight species inherently present in or released from the starting material, as also indicated by the corresponding no‐enzyme controls. Accordingly, the detected cyclic PA66‐derived species are not interpreted as quantitative evidence for newly generated polymer‐backbone cleavage. Instead, the PA66 experiments are interpreted primarily in terms of enzyme‐dependent transformation and redistribution of accessible PA‐derived species.

### 3.1 Single‐Enzyme Product Landscapes on Bulk Polyamides

To establish the soluble product profiles underlying the subsequent enzyme‐combination experiments, we first characterized the product landscapes generated by individual amidases on PA6 and PA66 under enzyme‐specific reaction conditions. These single‐enzyme hydrolysis profiles allowed characteristic soluble intermediates to be identified and provided the experimental basis for selecting complementary enzyme combinations.

Across all PA6 reactions, the extent of *ε*‐caprolactam release remained comparable between blank and enzyme‐treated controls (see Figure S10), confirming that none of the tested polyamidases catalyze lactam ring opening under these neutral conditions, consistent with previous observations [20, 21, 26]. This indicates that the lactam signal originates from pre‐existing low‐molecular‐weight species in the material rather than from enzymatic or spontaneous hydrolysis of the polymer backbone [34, 35]. Therefore, only linear hydrolysis products are shown graphically, whereas cyclic products are discussed only when pronounced changes are observed.

For PA6, distinct product distributions were observed depending on the enzyme employed (Figure 2A). NylA exhibited comparatively low activity and generated a mixture of linear 6‐AHA monomer and trimer, indicating limited turnover of the initially released oligomeric products. In contrast, NylB showed full conversion to the monomeric product 6‐AHA, consistent with its established preference for terminal amide‐bond hydrolysis in short linear nylon oligomers. Both NylC_p2_‐TS and NylC‐HP generated the linear 6‐AHA dimer as the main product, accompanied by smaller amounts of monomer. This product profile is in agreement with our previous studies on defined PA6 oligomers, where NylC variants preferentially cleaved internal amide bonds to yield dimeric intermediates [20]. The accumulation of the 6‐AHA dimer as the major product therefore identified a characteristic intermediate that could be targeted for further conversion by NylB.

**FIGURE 2:**
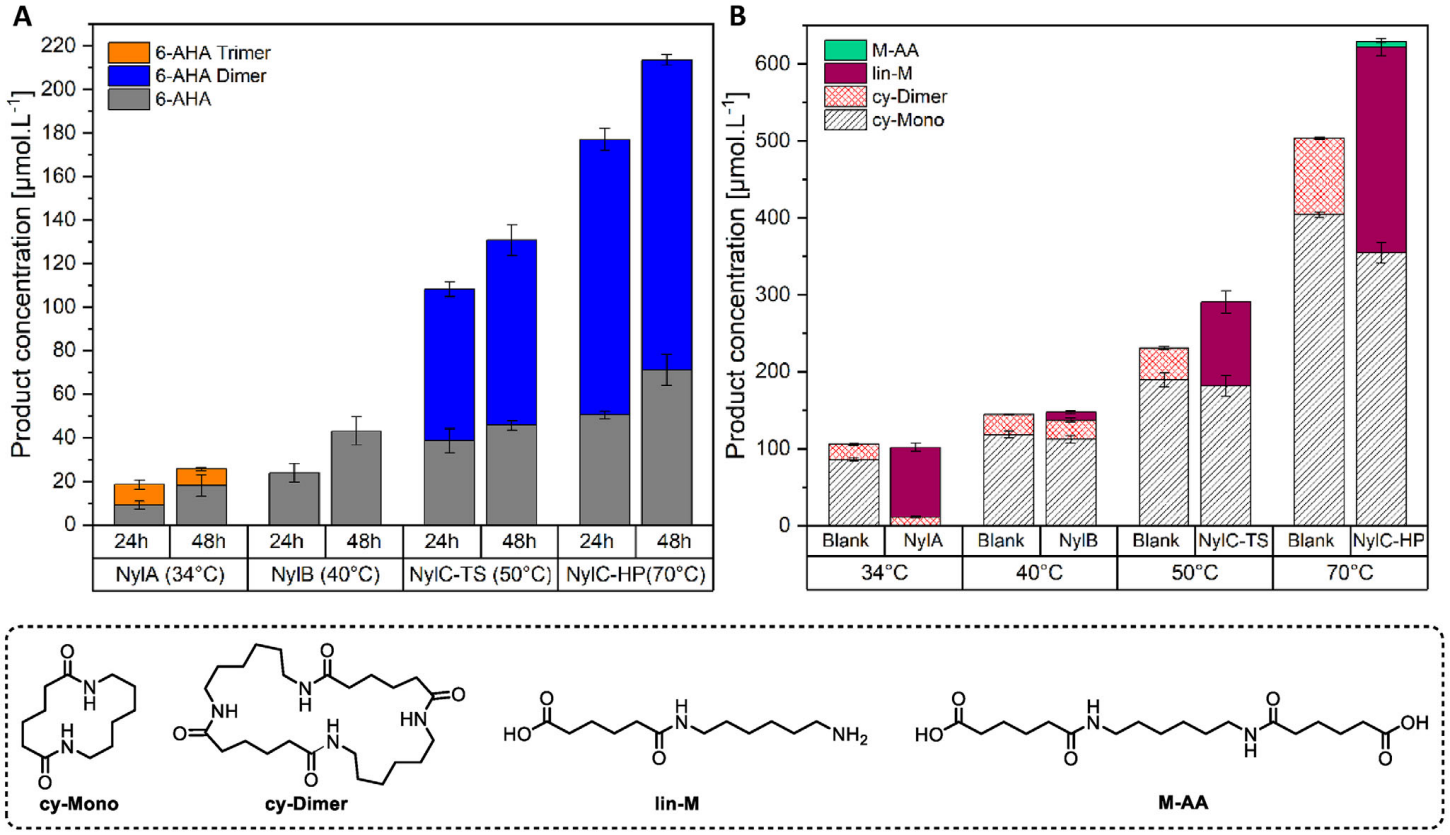
Single‐enzyme hydrolysis of bulk polyamide substrates. (A) Product distribution from PA6 film incubated with 1.1 µM enzyme. (B) Product distribution from PA66 granules incubated with 1.65 µM enzyme. Reactions were performed in 100 mM potassium phosphate buffer (pH 7.5, 150 mM NaCl) at enzyme‐specific reaction temperatures for 48 h. All assays were carried out in triplicate (*n* = 3); error bars represent the standard deviation of replicate measurements.

A similarly complementary product landscape was observed for PA66 after 48 h of hydrolysis (Figure 2B). Blank reactions consistently contained cyclic monomer and cyclic dimer species, reflecting extractable low‐molecular‐weight components inherently present in the polymer material [34, 35]. Consequently, changes in these species cannot by themselves be interpreted as quantitative evidence for polymer‐backbone cleavage. NylA selectively opened the cyclic monomer (cy‐Mono) to yield the corresponding linear PA66 repeat‐unit product (lin‐M), whereas NylC_p2_‐TS and NylC‐HP efficiently converted the cyclic dimer (cy‐Dimer) and subsequently hydrolyzed it further to lin‐M. In contrast, NylB showed only very limited activity and did not measurably convert cyclic intermediates. For the highly active NylC‐HP variant, formation of the diacid‐terminated fragment adipic acid–hexamethylenediamine–adipic acid (M–AA) was additionally observed, consistent with our previous results obtained using defined PA66 oligomer substrates [20]. Together, these complementary product distributions indicate that pairing NylC with NylA enables selective transformation of accessible PA66‐derived species toward linear downstream products.

### 3.2 Sequential Enzyme Treatments

To determine whether the characteristic soluble intermediates identified in the single‐enzyme experiments could be selectively converted by a complementary enzyme, we next investigated temporally separated enzyme treatments. This sequential design allows intermediate products generated by the first enzyme to accumulate before selective downstream conversion by the second enzyme, thereby providing a controlled framework to assess the proposed division of catalytic labor. For PA6, this approach is related to previously reported NylC/NylB combinations by Puetz et al. [26], while the present workflow provides time‐resolved insight into the downstream conversion of accumulated intermediates and extends the concept to PA66 depolymerization.

Based on the single‐enzyme kinetic experiments, 6‐AHA dimer accumulation from PA6 and cyclic monomer accumulation from PA66 both reached a clear plateau after 72 h (Figure S11). These plateau points were therefore selected as mechanistically informed switching points for sequential enzyme addition experiments. In all cases, bulk PA substrates were first hydrolyzed with NylC‐HP at 70°C for 72 h to promote soluble intermediate formation. Subsequently, the reaction temperature was reduced to 40°C for NylB or 34°C for NylA, respectively, and the second enzyme was added to selectively target the accumulated intermediates over an additional 48 h. To distinguish potential cooperative effects from simple catalyst replenishment, parallel enzyme‐refresh experiments were performed by adding fresh NylC‐HP after 72 h while maintaining the temperature at 70°C (Figure 3).

**FIGURE 3:**
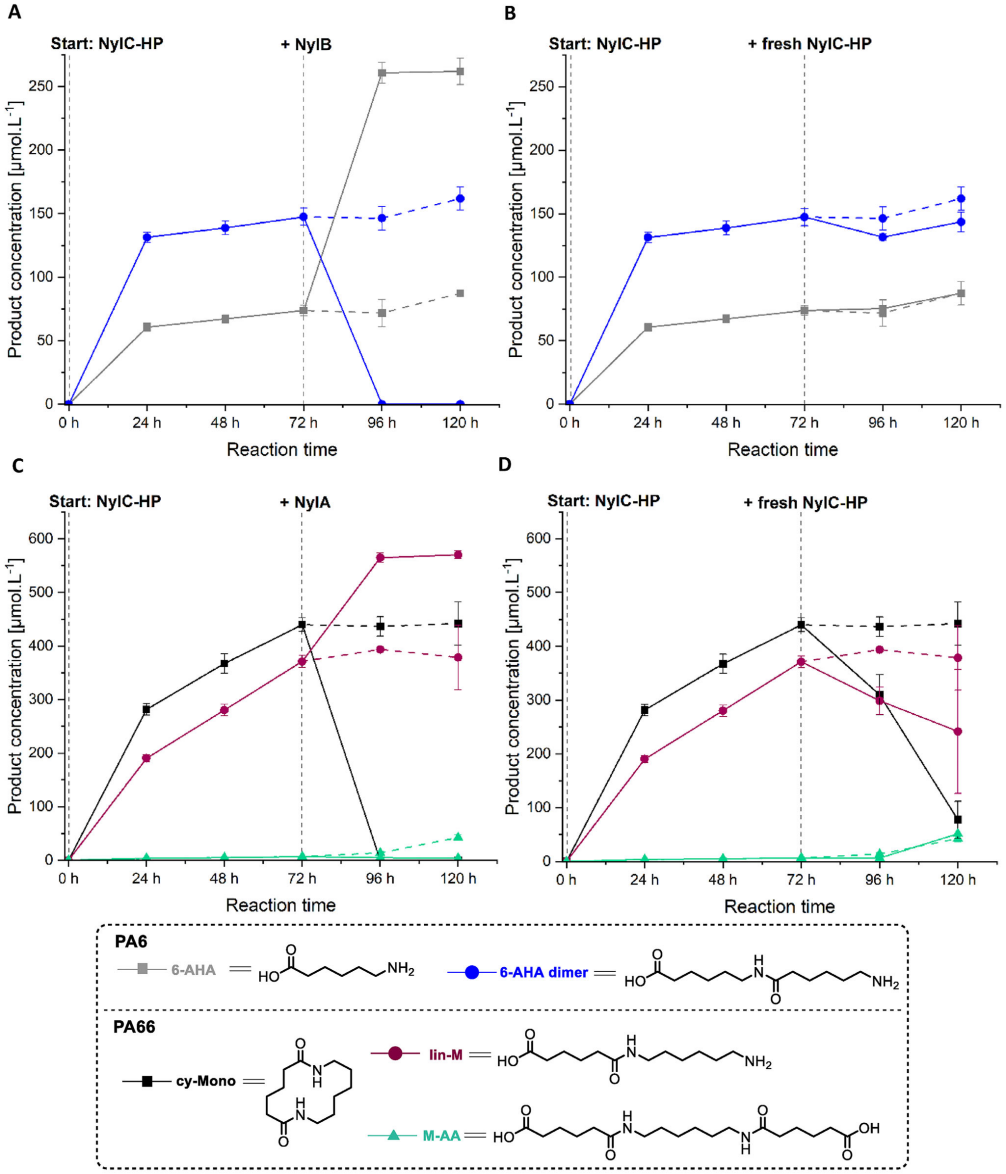
Time‐resolved product release from bulk polyamide substrates using sequential dual‐enzyme depolymerization strategies. (A) PA6 hydrolysis initiated with NylC‐HP at 70°C followed by the addition of NylB and incubation at 40°C after 72 h. (B) PA6 enzyme‐refresh experiment with addition of fresh NylC‐HP after 72 h and incubation was continued at 70°C. (C) PA66 hydrolysis initiated with NylC‐HP at 70°C followed by the addition of NylA and incubation at 34°C after 72 h. (D) PA66 enzyme‐refresh experiment with addition of fresh NylC‐HP after 72 h and incubation was continued at 70°C. The dashed lines represent the corresponding no‐addition control reactions, in which hydrolysis was continued with the initially added NylC‐HP only. All reactions were performed in 100 mM potassium phosphate buffer (pH 7.5, 150 mM NaCl). Error bars indicate the standard deviation of triplicate measurements (*n* = 3).

A pronounced downstream conversion effect was observed when NylB was added after 72 h to the PA6 hydrolysis reaction (Figure 3A). Within the following 24 h, the accumulated 6‐AHA dimer was completely depleted, while monomer concentration increased sharply to approximately 260 µmol L^−^^1^, corresponding to a 3.6‐fold increase relative to the no‐addition control. This transformation is consistent with the established dimer‐hydrolyzing activity of NylB [24]. Stoichiometrically, hydrolysis of one molecule of the 6‐AHA dimer can generate two molecules of 6‐AHA. However, the decrease in quantified dimer was not fully recovered as the corresponding increase in 6‐AHA equivalents, indicating that the soluble‐product balance was not quantitatively closed under these conditions. Accordingly, the experiment is interpreted as demonstrating downstream conversion of the accumulated dimer toward 6‐AHA rather than as a quantitative mass balance of PA6 hydrolysis.

Extending the reaction time beyond this point did not lead to further increases in monomer concentration, while the 6‐AHA dimer was no longer detectable under the applied analytical conditions, indicating establishment of a new product plateau. In contrast, the addition of fresh NylC‐HP after 72 h (Figure 3B) produced no substantial change relative to the no‐addition control. This restart experiment was included as a control to assess whether the observed plateau could be overcome by replenishment of the same catalyst, an approach previously reported for NylC‐mediated PA6 hydrolysis [21]. Under the present conditions, replenishment of NylC‐HP alone did not substantially increase soluble product formation. Expressed relative to the initial PA6 mass, the detected soluble products corresponded to yields below 1% under all investigated conditions. The NylC‐HP control yielded approximately 0.17% 6‐AHA and 0.59% 6‐AHA dimer, whereas after sequential NylB addition approximately 0.51% 6‐AHA was detected. Thus, despite the pronounced conversion of the accumulated dimer toward 6‐AHA, the overall amount of detectable soluble polymer‐derived material remained low. Together with the limited response to NylC‐HP replenishment, these low overall soluble product yields are consistent with restricted accessibility of the remaining hydrolyzable PA6 substrate. These observations suggest that further increases in overall PA6 conversion may therefore require improved polymer accessibility [21, 28], whereas selective downstream conversion of soluble oligomeric intermediates can increase the proportion of released monomeric products.

A similarly strong redistribution of soluble products was observed during PA66 hydrolysis after sequential addition of NylA (Figure 3C). Following the initial NylC‐HP phase, cyclic monomer (cy‐Mono) had accumulated as the dominant soluble species. Addition of NylA caused rapid depletion of the accessible cy‐Mono pool, accompanied by increased formation of lin‐M, with approximately 0.44% lin‐M and 0.45% cy‐Mono for the NylC‐HP control and approximately 0.63% lin‐M after sequential NylA addition. These observations are consistent with selective NylA‐mediated ring opening of soluble PA66‐derived cyclic species. Because cyclic species were also detected in the corresponding blank reactions, this transformation is interpreted as enzyme‐dependent redistribution of the accessible PA66‐derived product pool rather than as quantitative evidence for additional bulk‐polymer backbone cleavage. As discussed in the Methods section, cy‐Mono concentrations were estimated using a corrected surrogate calibration and should therefore be regarded as approximate. Accordingly, the quantified products do not constitute a closed molar balance of PA66 hydrolysis.

In comparison, re‐addition of fresh NylC‐HP also altered the soluble product distribution (Figure 3D), resulting in partial depletion of both cy‐Mono and lin‐M. A similar trend was previously observed for defined PA66 oligomers, where increased NylC‐HP loading accelerated depletion of lin‐M, whereas M–AA accumulated as a comparatively persistent downstream product [20]. The resistance of M–AA toward further enzymatic conversion might arise from steric and electrostatic effects associated with its terminal carboxylate groups. However, the observed depletion of cyclic and linear repeat‐unit species could not be quantitatively assigned to specific downstream products under the applied analytical conditions. In particular, no significant accumulation of adipic acid (AA) was observed (Figure S12). Calibration experiments established a practical detection limit of approximately 0.05 mM (Figure S13), suggesting that any AA formed remained below this threshold under the investigated conditions.

These results demonstrate that the established ring‐opening activity of NylA can be used to selectively redirect accessible cyclic PA66‐derived species toward linear downstream products during sequential enzyme treatment. Collectively, the sequential experiments show that the complementary substrate preferences identified in the single‐enzyme assays can be exploited to selectively convert soluble PA‐derived intermediates and alter the resulting product distributions.

### 3.3 One‐Pot Two Enzyme Systems

Following the sequential experiments, we next investigated whether one‐pot two‐enzyme systems could continuously couple primary polymer depolymerization with downstream intermediate conversion, thereby minimizing soluble product accumulation throughout the reaction without requiring temperature switching or separate process stages. For this purpose, NylC_p2_‐TS was selected as the NylC variant because it retains substantial activity at temperatures closer to the operating ranges of NylB and NylA [21, 32, 33]. This enabled evaluation of concurrent enzyme activity under temperature‐compatible conditions. Corresponding temperature‐matched no‐enzyme controls are provided in Figure S14 for PA6 and in Figure 2B for PA66.

Under these temperature‐compatible conditions, PA6 hydrolysis at 40°C showed a pronounced increase in monomer release when NylC_p2_‐TS/NylB mixtures were applied for 48 h (Figure 4A). Across the investigated enzyme ratios, the mixed‐enzyme reactions consistently shifted the soluble product distribution toward 6‐AHA relative to either enzyme alone. Although the numerically highest 6‐AHA concentration was observed at a NylC_p2_‐TS/NylB ratio of 75:25, differences among the neighboring mixture ratios were comparatively small within the experimental variability. These results indicate that inclusion of NylB promotes downstream conversion of the soluble 6‐AHA dimer generated during NylC_p2_‐TS treatment.

**FIGURE 4:**
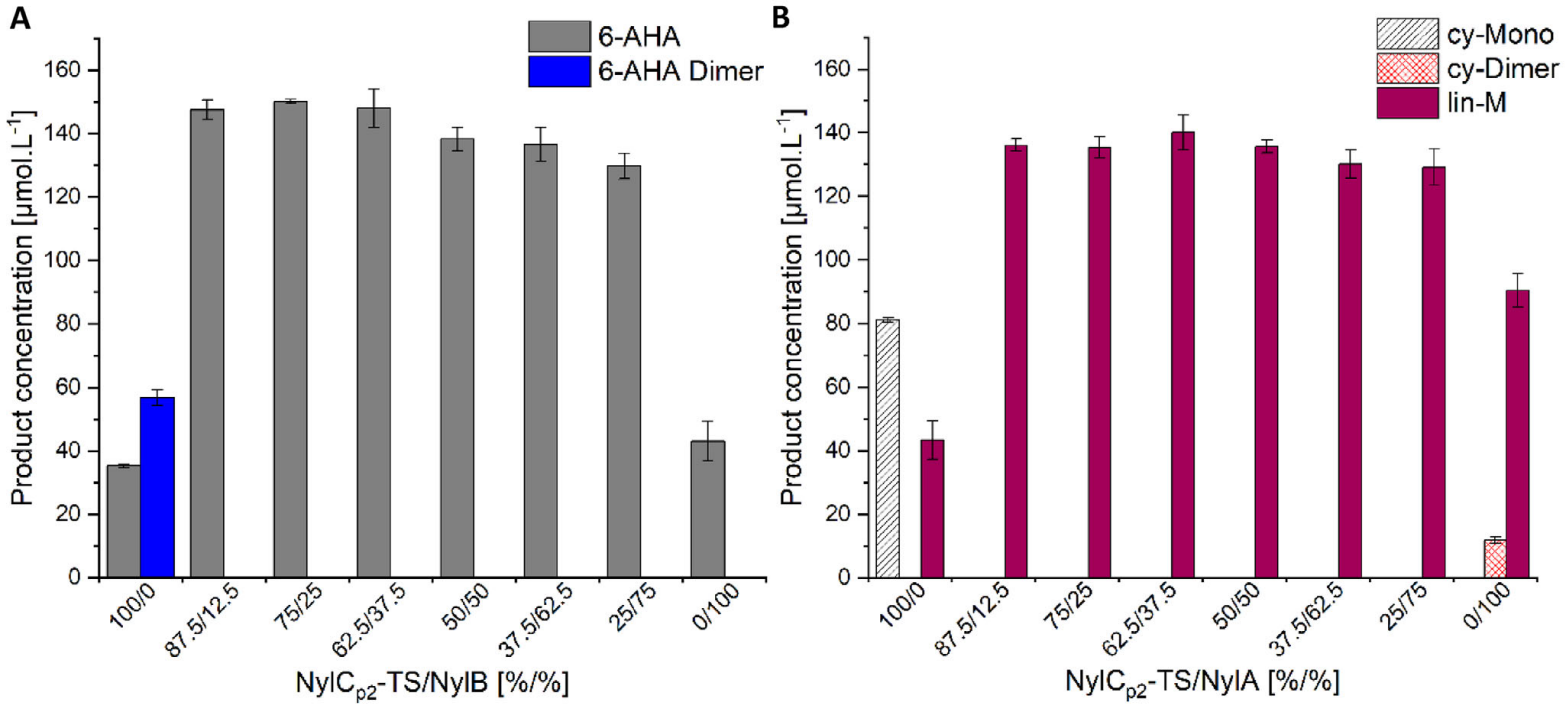
One‐pot two‐enzyme systems for enzymatic treatment of bulk polyamide substrates under temperature‐compatible conditions. (A) Product release from PA6 film after 48 h hydrolysis at 40°C using mixtures of NylC_p2_‐TS and NylB at varying enzyme ratios. (B) Product release from PA66 granules after 48 h hydrolysis at 34°C using mixtures of NylC_p2_‐TS and NylA at varying enzyme ratios. All reactions were performed in 100 mM potassium phosphate buffer (pH 7.5, 150 mM NaCl). The total enzyme concentration was kept constant across all mixtures at 1.1 µM for PA6 and 1.65 µM for PA66, corresponding to substrate‐normalized total enzyme loadings of 0.164 µmol enzyme g^−1^ PA6 and 0.075 µmol enzyme g^−1^ PA66, respectively. Error bars represent the standard deviation of triplicate measurements (*n* = 3).

Notably, the one‐pot NylC_p2_‐TS/NylB system produced approximately 150 µmol L^−1^ 6‐AHA at 40°C, exceeding the apparent total soluble product concentration obtained with NylC_p2_‐TS alone even at 50°C (Figure 2A). However, when the dimeric products are expressed in 6‐AHA equivalents, the total soluble product release remains broadly comparable between the NylC_p2_‐TS/NylB mixtures and the corresponding single‐enzyme controls. Thus, the principal effect of NylB was not a substantial increase in overall soluble product release, but redistribution of the soluble product pool toward monomer formation.

A comparable redistribution of soluble products was observed during PA66 hydrolysis at 34°C in the one‐pot NylC_p2_‐TS/NylA system (Figure 4B). Relative to the individual enzymes, the mixed‐enzyme reactions showed increased formation of lin‐M together with reduced abundance of accessible cyclic PA66‐derived species, consistent with NylA‐mediated ring opening. However, differences between the investigated enzyme ratios remained comparatively small within the experimental variability, suggesting that the resulting product distribution was not strongly dependent on the precise NylC_p2_‐TS/NylA ratio under the applied conditions. Instead, as observed for PA6, the amount of detectable soluble product remained low, consistent with limited release of hydrolyzable species from the bulk PA66 substrate. Similar to the sequential experiments, the PA66 one‐pot two‐enzyme reaction is therefore interpreted primarily in terms of enzyme‐dependent redistribution of accessible soluble products rather than quantitative bulk‐polymer conversion. Nonetheless, the results demonstrate that NylA can redirect accessible cyclic PA66‐derived species toward the linear repeat‐unit product under mild, temperature‐compatible conditions.

The ability to combine complementary amidases at 34°C–40°C demonstrates that downstream intermediate conversion can be achieved under temperature‐compatible one‐pot conditions. However, the low overall soluble product yields and the plateau behavior observed across the investigated systems indicate that limited substrate accessibility remains an important constraint on bulk PA depolymerization. Thus, the principal advantage of the temperature‐compatible one‐pot two‐enzyme system lies in coordinated downstream intermediate conversion and control of product distribution rather than in overcoming the intrinsic substrate‐accessibility limitation of bulk PA hydrolysis. In the future, combining such cooperative catalyst systems with pretreatment strategies that improve polymer accessibility, such as particle size reduction or other approaches that reduce structural barriers to enzyme access, may provide an effective route toward higher overall depolymerization efficiencies while retaining control over the resulting product distribution.

## 4 Conclusion

In this study, we demonstrate that complementary amidases can be combined to control downstream intermediate conversion and product distribution during enzymatic treatment of bulk PA materials. Sequential enzyme treatments showed that characteristic soluble intermediates generated or accumulated during NylC‐mediated hydrolysis can be selectively converted by complementary enzymes. For PA6, NylB converted the accumulated 6‐AHA dimer toward 6‐AHA, consistent with its established activity toward short linear PA6 oligomers. For PA66, sequential coupling of NylC and NylA showed that accessible cyclic intermediates can be selectively ring‐opened and redirected toward the linear repeat‐unit product. Together, these experiments provide mechanistic evidence that complementary substrate preferences can be exploited to control the downstream conversion of soluble intermediates during enzymatic treatment of bulk PA substrates.

This concept was further translated into temperature‐compatible one‐pot two‐enzyme systems using NylC_p2_‐TS in combination with NylB or NylA. Under these conditions, downstream intermediate conversion could be achieved under milder conditions without requiring temperature switching between catalytic stages. However, the low overall soluble product yields indicate that limited substrate accessibility remains a major constraint on bulk PA depolymerization. Thus, the principal benefit of the two‐enzyme systems lies in controlling product distribution and selectively directing soluble intermediates toward desired products, rather than overcoming limitations in overall substrate conversion. Future strategies may therefore benefit from integrating complementary enzyme systems with pretreatment approaches that increase polymer accessibility to achieve higher overall conversion.

More broadly, these findings highlight catalyst matching as a complementary strategy to conventional protein engineering approaches based on mutagenesis and thermostabilization. The demonstrated compatibility of amidases with distinct substrate preferences provides a conceptual basis for designing multienzyme systems in which soluble intermediates generated during polymer hydrolysis are selectively converted by downstream catalysts. Such systems could in future be further developed through approaches such as enzyme fusions or spatially organized multienzyme assemblies to facilitate sequential catalytic steps. Overall, this work provides mechanistic insight into how complementary enzymatic activities can be coordinated during PA degradation and supports the further development of selective, low‐temperature multienzyme strategies for PA recycling.

## Conflicts of Interest

The authors declare no conflicts of interest.

## Supporting information




**Supporting File**: mabi70274‐sup‐0001‐SuppMat.pdf.

## Data Availability

The data that support the findings of this study are available from the corresponding author upon reasonable request.
