# Supplementary material for: Cooperative Amidase Systems Control Product Selectivity During Enzymatic Nylon Depolymerization
Source: Macromol Biosci. 2026 Oct 15;26(10):e70274. doi: 10.1002/mabi.70274 (PMC13672674; doi:10.1002/mabi.70274)
Supplement: Supplementary file 1 [file MABI-26-e70274-s001.pdf]

Supporting Information

Cooperative Amidase Systems Control Product Selectivity during Enzymatic Nylon Depolymerization

Sukmanita Dewi[1], Nico Bäse[2], Alan Wypych[2], Hendrik Puetz[3], Christoph Janknecht[3], Ulrich Schwaneberg[3], Wolfgang R. Streit[2], Laura Hartmann[1,4*]

[1]Institute of Macromolecular Chemistry, Albert-Ludwigs-University of Freiburg, Freiburg, Germany

[2]Department of Microbiology and Biotechnology, Biocenter Klein Flottbek, University of Hamburg, Hamburg, Germany

[3]Institute of Biotechnology, RWTH Aachen University, Aachen, Germany

[4]Freiburg Materials Research Center, Albert-Ludwigs-University of Freiburg, Freiburg, Germany

[*]Corresponding author, laura.hartmann@makro.uni-freiburg.de, Institute of Macromolecular Chemistry, Albert-Ludwigs-University of Freiburg, Freiburg, Germany

Supporting Methods

Expression Plasmid Construction

Genes encoding NylA, NylB, and $NylC_{p2}$-TS ($NylC_{p2}$-GYAQ) were cloned into pET21a(+) expression vectors and expressed with C-terminal polyhistidine tags, following the procedure reported by Wypych *et al*.[1] The engineered variant NylC-HP ($NylC_{p2}$-$TS^{F134W/D304M/R330A}$) was constructed and cloned according to the procedure reported by Puetz *et al*.[2]

Recombinant Protein Production

- NyA, NylB, and $NylC_{p2}$-TS

Recombinant expression of NylA, NylB, and $NylC_{p2}$-TS was carried out in *Escherichia coli* T7 Express lysY/Iq (New England Biolabs, USA) harboring the corresponding pET21a(+) expression constructs, following Wypych *et al*.[1,3,4] Cells were cultivated in LB medium supplemented with 100 µg mL^{-1} ampicillin under constant aeration at 37°C. Upon reaching an optical density (OD_{600}) of approximately 0.6, protein expression was induced with 1 mM IPTG, and cultures were incubated overnight at 22°C.

Cells were harvested by centrifugation (8,000 rpm, 15 min, 4°C; Sorvall RC 6+, Thermo Scientific, USA) and resuspended in NPI-10 lysis buffer (50 mM NaH_2PO_4, 300 mM NaCl, 10 mM imidazole, pH 8.0). Cell disruption was performed using a French pressure cell press (American Instrument Company, USA) at 1300 psi.

- NylC-HP

Recombinant expression of the engineered variant NylC-HP was performed in *Escherichia coli* BL21(DE3) according to Puetz *et al*.[2] Cultures were grown at 37°C to an OD_{600} of approximately 0.6, induced with 0.1 mM IPTG, and incubated for 20 h at 18°C.

Cells were harvested by centrifugation (10,000 rpm, 20 min, 4°C; Eppendorf 5810R, Eppendorf SE, Germany) and resuspended in lysis buffer (50 mM NaH_2PO_4, pH 8.0, 300 mM NaCl) supplemented with lysozyme (1.5 mg mL^{-1}). After incubation for 30 min at 37°C, cells were lysed by sonication (Vibra-Cell VCX130, Sonics & Materials Inc., USA; 5 × 30 s pulses at 60% amplitude with 30 s cooling intervals).

Protein Purification

- NyA, NylB, and $NylC_{p2}$-TS

Crude cell extracts were clarified by centrifugation (20,000 rpm, 20 min, 4°C), and the resulting supernatants were purified by immobilized metal affinity chromatography (IMAC) using Ni–NTA agarose (Macherey-Nagel GmbH, Germany) for polyhistidine-tagged proteins. After washing, with NPI-20 (50 mM NaH_2PO_4, 300 mM NaCl, 20 mM imidazole, pH 8.0), proteins were eluted using NPI-250 (50 mM NaH_2PO_4, 300 mM NaCl, 250 mM imidazole, pH 8.0) buffer and afterwards dialyzed and concentrated with sodium phosphate (NaPi) buffer (0.1 M, pH 8.0) using Vivaspin 20 concentrator columns with 10 kDa PES filter (Sartorius, Göttingen, Germany). Protein concentration was determined using a nanodrop NanoPhotometer© NP80 (Implen GmbH, München, Germany).

- NylC-HP

The lysate was clarified by centrifugation (10,000 rpm, 30 min, 4°C; Eppendorf 5810R, Germany) and filtered through a 0.45 µm syringe filter. NylC-HP was purified by Ni–IDA affinity chromatography (Macherey-Nagel GmbH, Germany). After washing with lysis buffer, the protein was eluted using elution buffer (50 mM NaH_2PO_4, pH 8.0, 300 mM NaCl, 250 mM imidazole).

Buffer exchange into storage buffer (50 mM Bicine, pH 8.0, 100 mM NaCl) was performed using PD-10 desalting columns (Cytiva, USA), followed by concentration using 10 kDa MWCO Amicon Ultra centrifugal filters (Merck, Germany). The purified protein was snap-frozen in liquid nitrogen and stored at −20°C. Protein purity was assessed by SDS–PAGE, and concentrations were determined using the Bradford assay.[5]

Amino Acid Sequences of Applied Enzymes

Table T1. Overview of amidase variants used in this study.

Enzyme	Variant	Amino acid substitutions	Reference
NylA	WT	–	Kinoshita et al.[6]
NylB	WT	–	Negoro et al.[4]
$NylC_{p2}$-TS	thermostabilized	D122G/H130Y/D36A/E263Q	Negoro et al.[7]
NylC-HP	engineered variant	D122G/H130Y/D36A/E263Q/F134W/D304M/ R330A	Puetz et al.[2]

Introduced mutations are underlined and in bold.

- NylA

MSKVDLWQDATAQAELVRSGEISRTELLEATIAHVQAVNPEINAVIIPLFEKARRESELASGPFAGVPYLLKDLTVVS
QGDINTSSIKGMKESGYRADHDAYFVQRMRAAGFVLLGKTNTPEMGNQVTTEPEAWGATRNPWNLGRSVGGSS
GGSGAAVAAALSPVAHGNDAAGSVRIPASVCGVVGLKPTRGRISPGPLVTDSDNVAGAAHEGLFARSVRDIAALLD
VVSGHRPGDTFCAPTASRPYAQGISENPGSLRVGVLTHNPVGDFALDPECAAAARGAAAALAALGHDVNDAYPEA
LGDRSFLKDYSTICDVAIAREIERNGELIGRPLTEDDVEWTSWEMVKRADQVTGRAFAACVDELRYYAGKVERWW
EAGWDLLILPTVTRQTPEIGELMLAKGTDLEGRQSAFISGSLQMLAFTVPFNVSGQPAISLPIGMSSDGMPIGVQIV
AAYGREDLLLQVAAQLEGALPWVARRPQLLNPSRKIPAA

- NylB

MNARSTGQHPARYPGAAAGEPTLDSWQEAPHNRWAFARLGELLPTAAVSRRDPATPAEPVVRLDALATRLPDLE
QRLEETCTDAFLVLRGSEVLAEYYRAGFAPDDRHLLMSVSKSLCGTVVGALIDEGRIDPAQPVTEYVPELAGSVYDGP
SVLQVLDMQISIDYNEDYVDPASEVQTHDRSAGWRTRRDGDPADTYEFLTTLRGDGGTGEFQYCSANTDVLAWIV

ERVTGLRYVEALSTYLWAKLDADRDATITVDQTGFGFANGGVSCTARDLARVGRMMLDGGVAPGGRVVSQGWV
ESVLAGGSREAMTDEEGFTSAFPEGSYTRQWWCTGNERGNVSGIGIHGQNLWLDPRTDSVIVKLSSWPDPDTRH
WHGLQSGILLDVSRALDAV

- NylC$_{p2}$-TS

MMHHHHHHGA[1]GANTTPVHALTDIDGGIAVDPAPRLAGPPVFGGPGN**A**AFDLAPVRSTGREMLRFDFPGVSIGA
AHYEEGPTGATVIHIPAGARTAVDARGGAVGLSGGYDFNHAICLAGGAGYGLEAGAGVS**G**ALLERLE**Y**RTGFAELQ
LVSSAVIYDFSARSTAVYPDKALGRAALEFAVPGEFPQGRAGAGMSASAGKVDWDRTEITGQGAAFRRLGDVRILA
VVVPNPVGVIVDRAGTVVRGNYDAQTGVRRHPVFDYQEAFAEQVPPVT**Q**AGNTTISAIVTNVRMSPVELNQFAK
QVHSSMHRGIQPFHTDMDGDTLFAVTTDEIDLPTTPGSSRGRLSVNATALGAIASEVMWDAVLEAGK*

- NylC-HP

MMHHHHHHGA[1]GANTTPVHALTDIDGGIAVDPAPRLAGPPVFGGPGN**A**AFDLAPVRSTGREMLRFDFPGVSIGA
AHYEEGPTGATVIHIPAGARTAVDARGGAVGLSGGYDFNHAICLAGGAGYGLEAGAGVS**G**ALLERLE**Y**RTG**W**AEL
QLVSSAVIYDFSARSTAVYPDKALGRAALEFAVPGEFPQGRAGAGMSASAGKVDWDRTEITGQGAAFRRLGDVRIL
AVVVPNPVGVIVDRAGTVVRGNYDAQTGVRRHPVFDYQEAFAEQVPPVT**Q**AGNTTISAIVTNVRMSPVELNQFA
KQVHSSMHRGIQPFHT**M**MDGDTLFAVTTDEIDLPTTPGSSRG**A**LSVNATALGAIASEVMWDAVLEAGK*

Differential Scanning Calorimetry (DSC)

Differential scanning calorimetry measurements were performed using a DSC 204 F1 instrument (NETZSCH Gerätebau GmbH, Selb, Germany). Samples were heated from 25 to 300°C at a constant heating rate of 10°C min^{-1}. After an isothermal hold at 300°C for 2 min, the samples were cooled to 25°C at 10°C min^{-1}, held at 25°C for 5 min, and subsequently heated again to 300°C at 10°C min^{-1}.

The degree of crystallinity χ_c of the untreated PA substrates was determined from the melting enthalpy obtained during the first heating scan according to Equation 1:

$$\chi_c(\%) = \frac{\Delta H_m}{\Delta H_m^{\circ}} * 100 \quad (1)$$

where ΔH_m is the experimentally determined melting enthalpy and $\Delta H°_m$ is the melting enthalpy of the corresponding 100% crystalline polymer. Values of $\Delta H°_m$ = 230 J g^{-1} for PA6 and 226 J g^{-1} for PA66 were used.[8,9]

Determination of the Cyclic Monomer PA66 Response Correction Factor

Cyclic PA66 monomer (cy-Mono) concentrations were quantified using the calibration curve of the corresponding linear monomer (lin-M) as a surrogate standard because an authentic cy-Mono standard was not available. However, comparison of cy-Mono depletion and lin-M formation during enzymatic ring-opening reactions catalyzed by NylA revealed substantial differences in detector response between cyclic and linear species under the applied RP-HPLC conditions.

To account for this effect, an empirical correction factor was derived from the relationship between the decrease in the cy-Mono signal and the corresponding formation of lin-M during NylA-mediated ring opening. Based on these measurements, a correction factor of 0.45 was applied to all cy-Mono quantifications presented in this study. Because this correction was derived from an enzymatic conversion experiment rather than from an authentic cy-Mono standard, the resulting cy-Mono concentrations should be regarded as semi-quantitative estimates and were not used to establish stoichiometric conversion or a closed molar balance.

High-Performance Liquid Chromatography (HPLC) Analysis of Adipic Acid

To quantify adipic acid (AA) formed during enzymatic depolymerization of PA66, degradation samples were analyzed using a Shimadzu Prominence LC system equipped with a C18 reversed-phase column (NUCLEOSIL 120-10 C18, Macherey-Nagel GmbH & Co. KG, Germany). Detection was performed using a photodiode array detector (SPD-M20A, Shimadzu, Japan) at 220 nm.

The mobile phase consisted of 0.1% sulfuric acid in water (mobile phase A) and acetonitrile (mobile phase B). Samples (10 µL injection volume) were analyzed under isocratic conditions using 10% mobile phase B at a constant flow rate of 1.0 mL min^{-1} over a total runtime of 20 min. Under these conditions, AA eluted at a retention time (t_R) of 5.2 min.

Supporting Figures

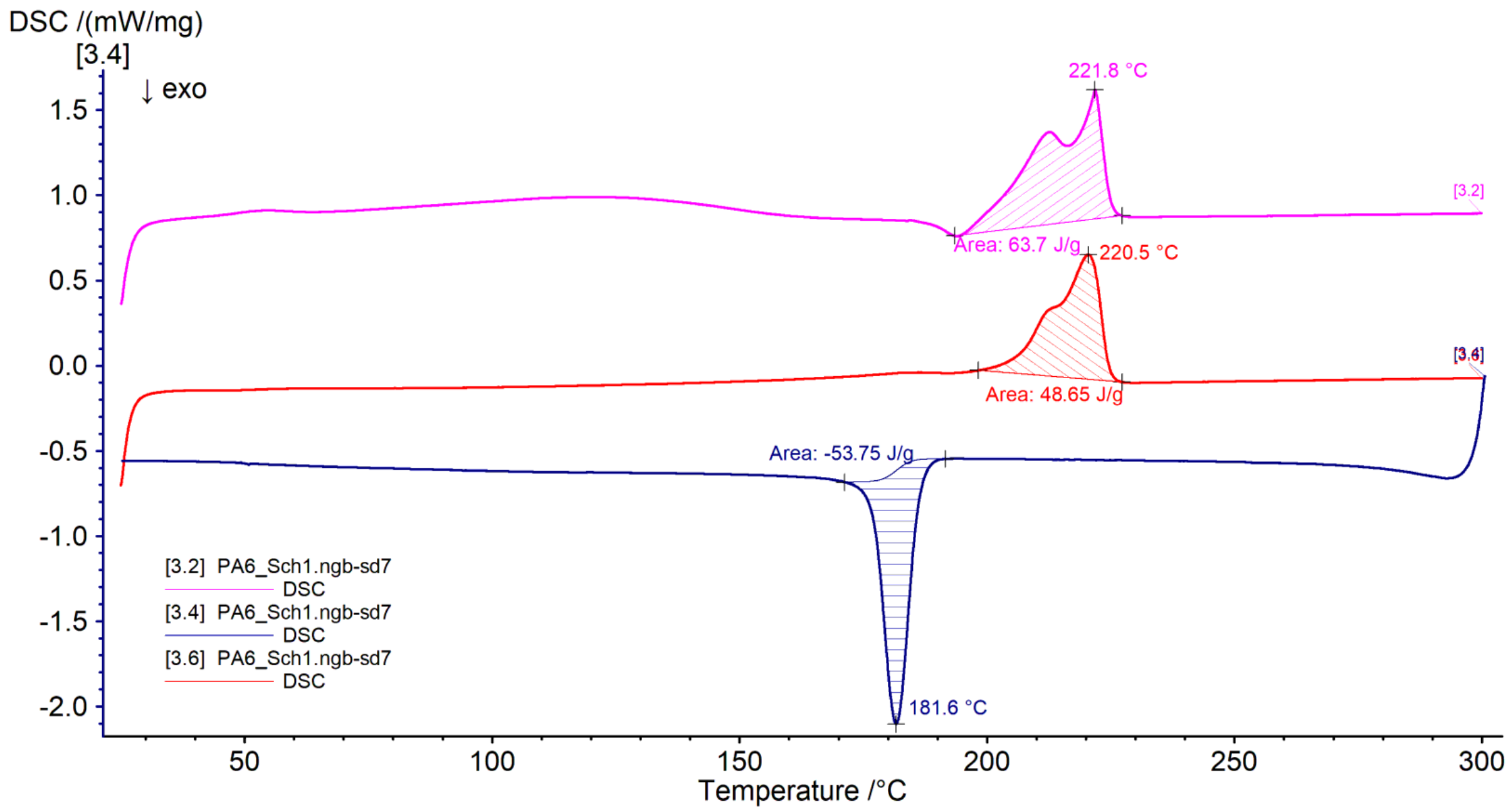

Figure S1. DSC thermogram of the PA6 film used in this study. The crystallinity was determined as 27.7%.

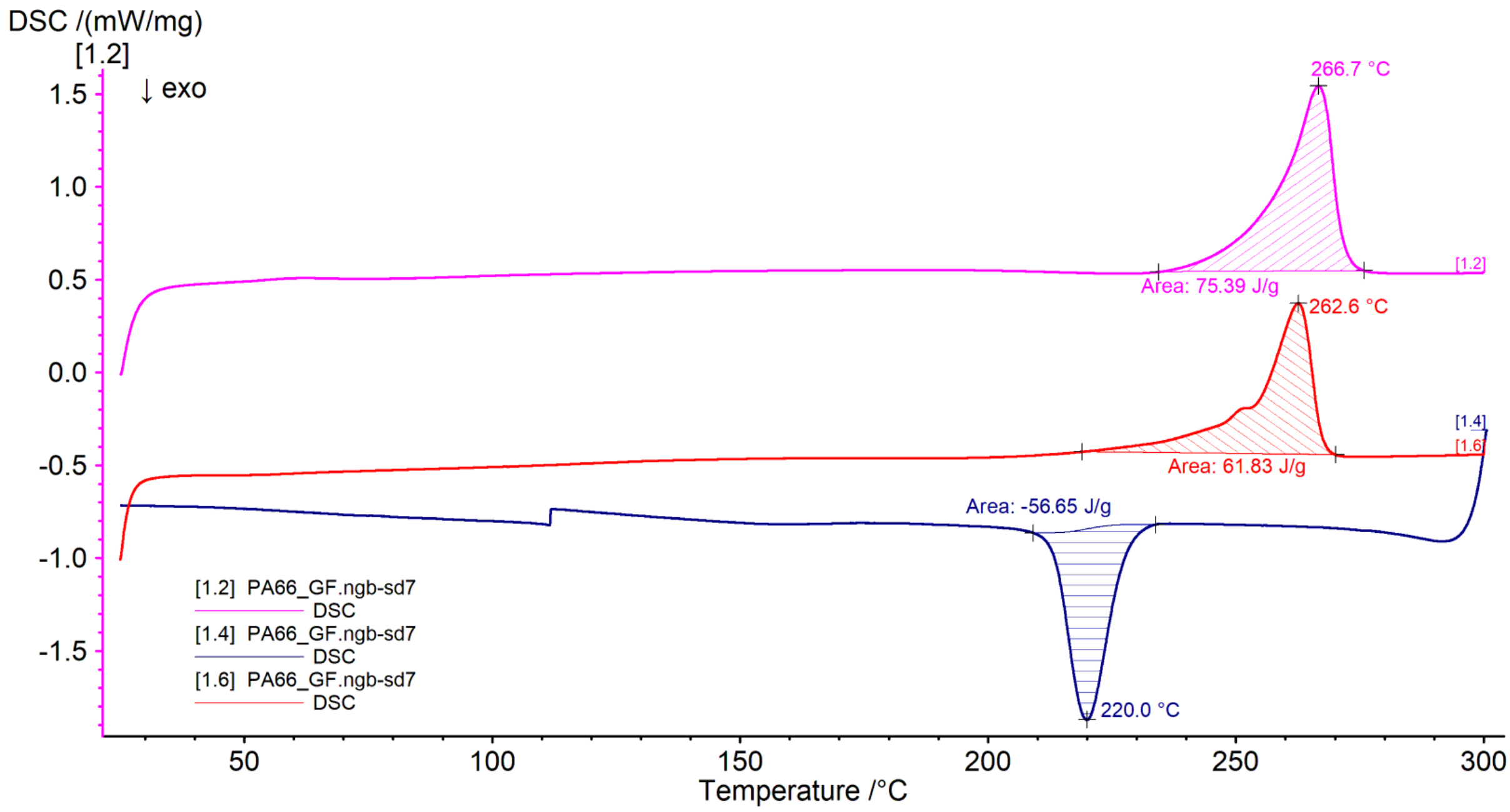

Figure S2. DSC thermogram of the PA66 granules used in this study. The crystallinity was determined as 33.4%.

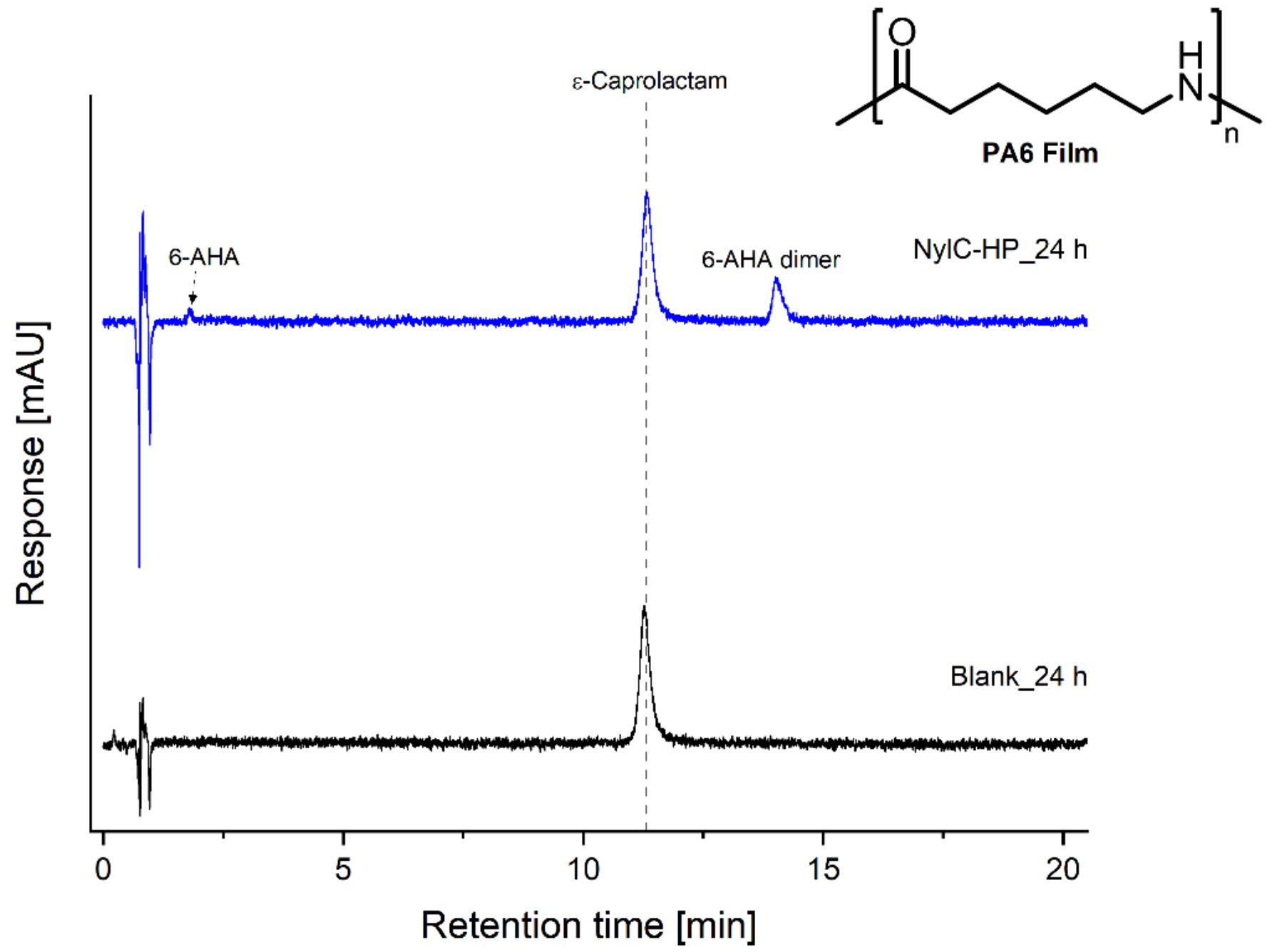

Figure S3. RP-HPLC chromatograms of PA6 film incubated without enzyme (blank, black) or with NylC-HP (blue) at 70°C for 24 h. Peaks were assigned to 6-AHA monomer (1.9 min), *ε*-caprolactam (11.8 min), and 6-AHA dimer (14.3 min). Chromatograms were recorded using an isocratic hold at 100% water for 5 min, followed by a linear gradient from 0 to 10% acetonitrile over 15 min with UV detection at 205 nm.

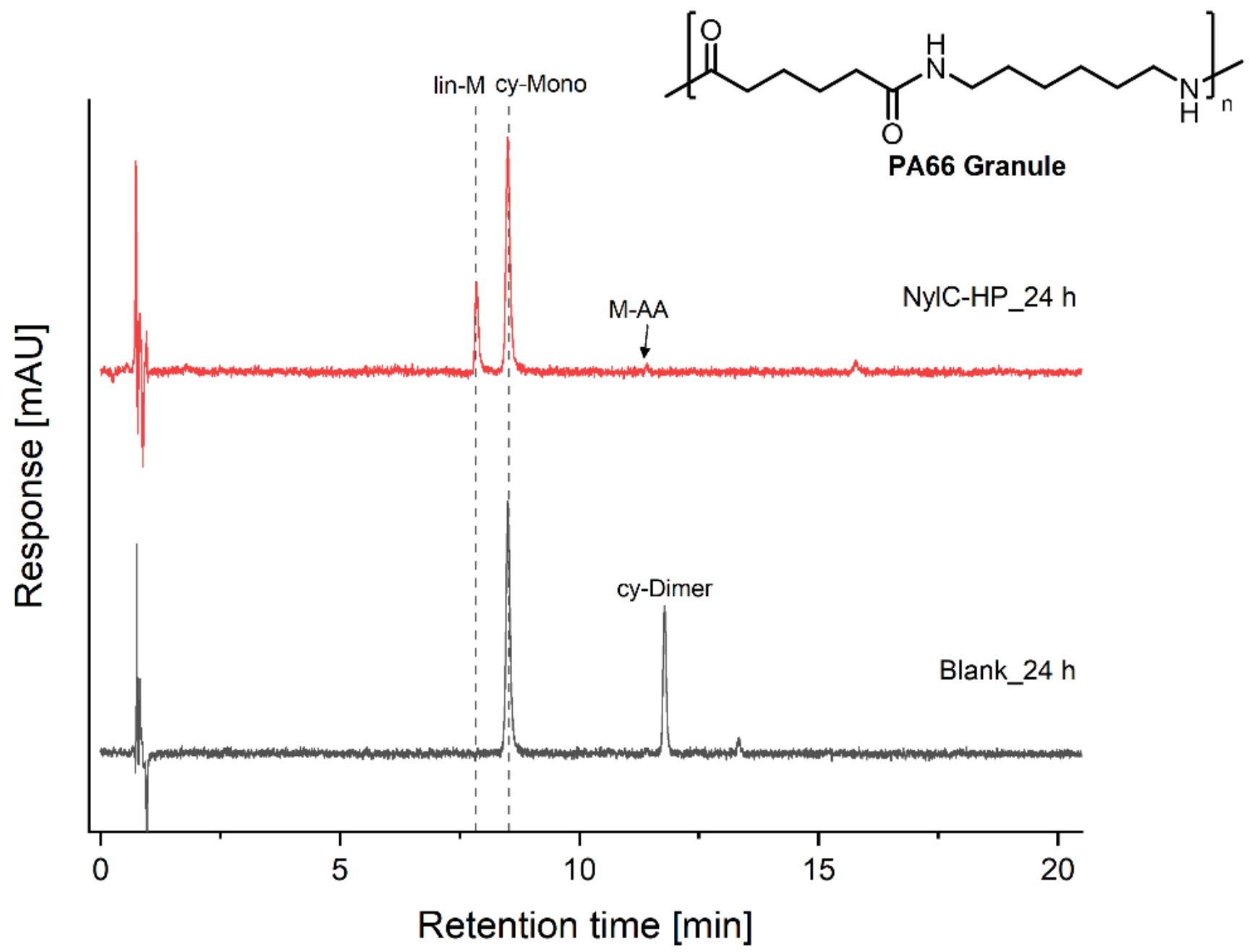

Figure S4. RP-HPLC chromatograms of PA66 granules incubated without enzyme (blank, black) or with NylC-HP (red) at 70°C for 24 h. Peaks were assigned to linear monomeric product (lin-M, 7.9 min), cyclic monomer (cy-Mono, 8.6 min), diacid-terminated fragment M−AA (11.5 min), and cyclic dimer (cy-Dimer, 11.8 min), as indicated. Chromatograms were recorded using an isocratic hold at 100% water for 3 min, followed by a linear gradient from 0 to 50% acetonitrile over 15 min with UV detection at 205 nm.

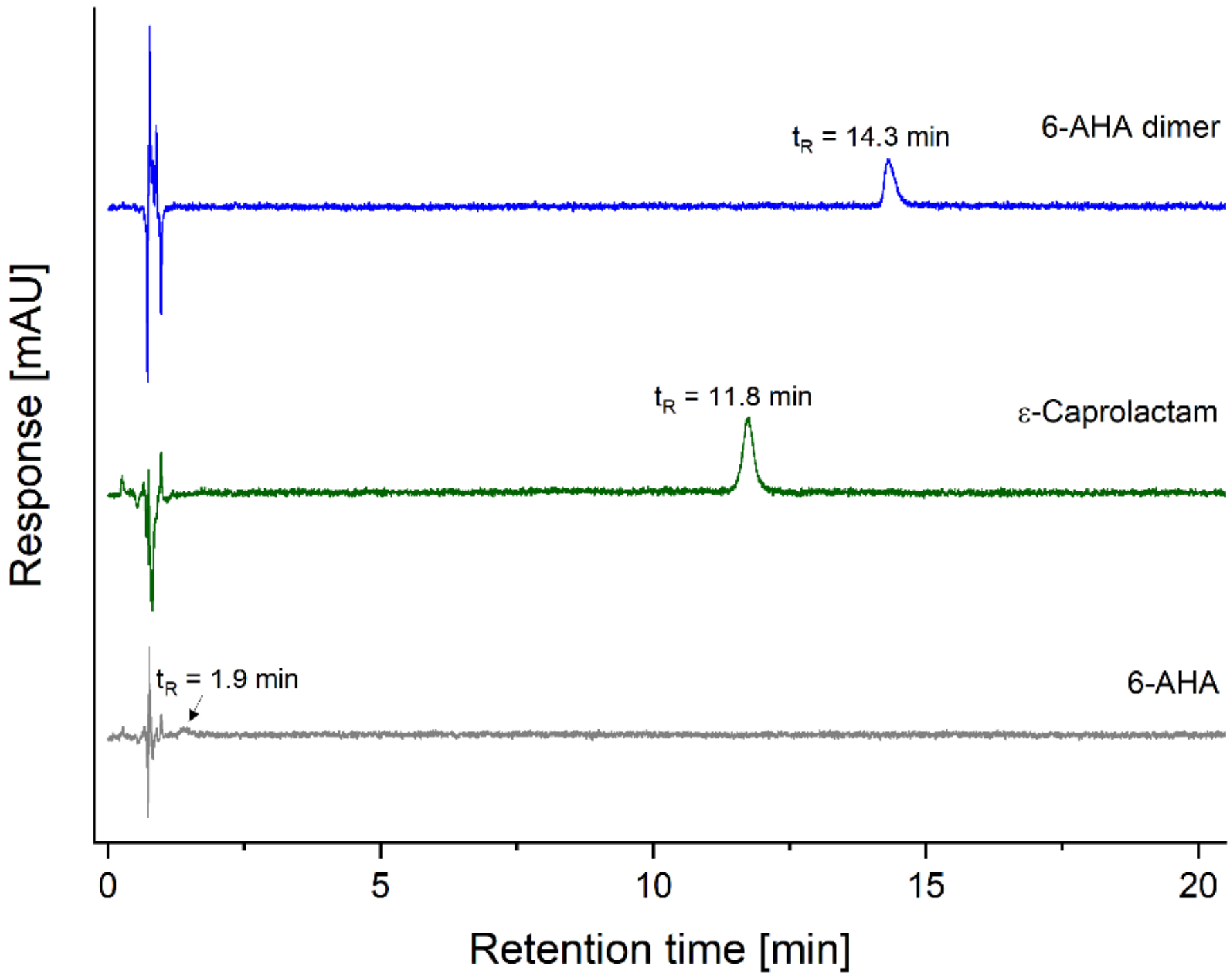

Figure S5. RP-HPLC method for the detection of PA6 film degradation products. Chromatograms of 6-aminohexanoic acid (6-AHA) monomer (t_R = 1.9 min), *ε*-caprolactam (t_R = 11.8 min), and 6-AHA dimer (t_R = 14.3 min), each at 0.1 mM. Chromatograms were recorded using an isocratic hold at 100% water for 5 min, followed by a linear gradient from 0 to 10% acetonitrile over 15 min with UV detection at 205 nm.

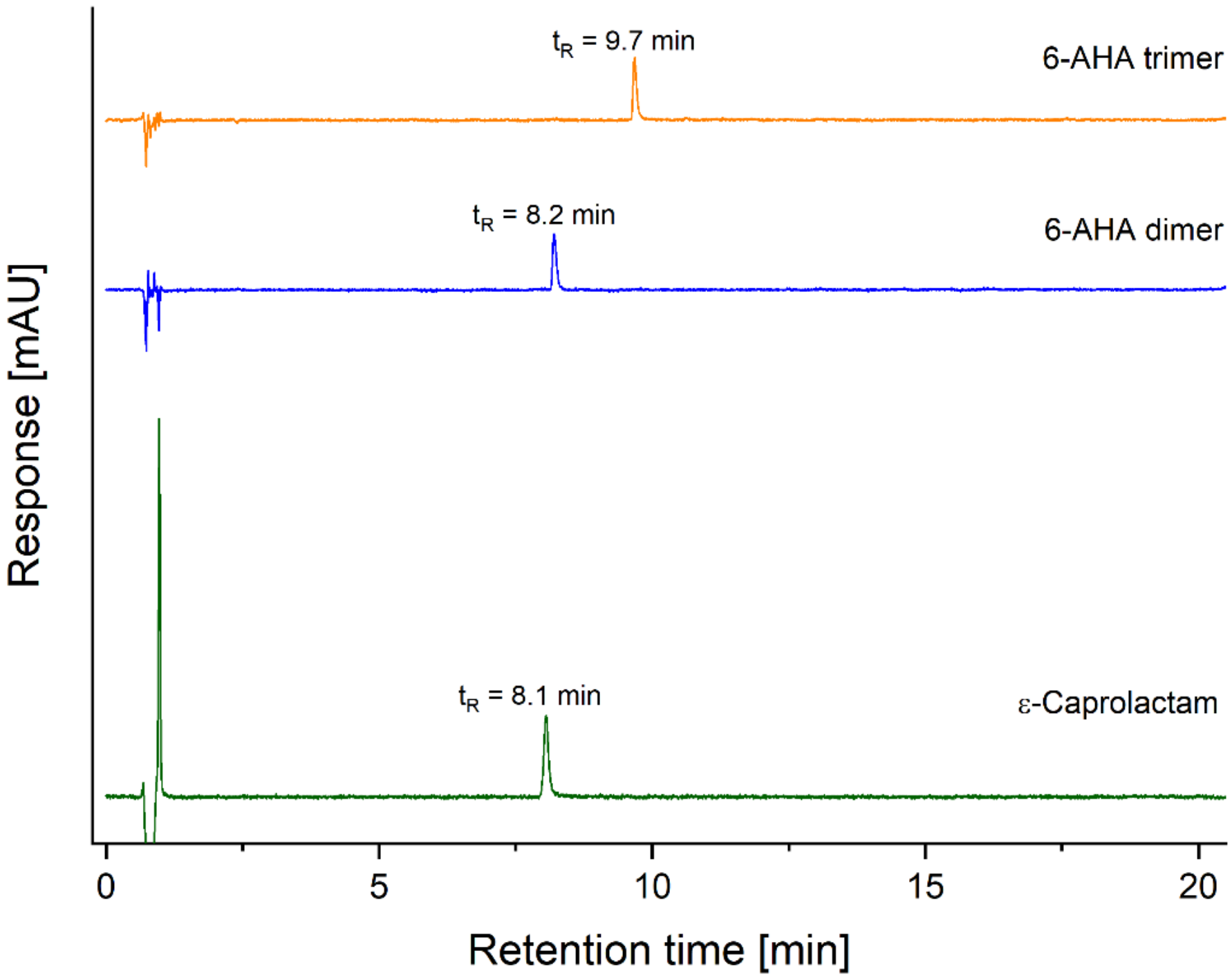

Figure S6. RP-HPLC method for the detection of higher PA6-derived degradation products. Chromatograms of *ε*-caprolactam (t_R = 8.1 min), 6-AHA dimer (t_R = 8.2 min), and 6-AHA trimer (t_R = 9.7 min), each at 0.1 mM. Chromatograms were recorded using an isocratic hold at 100% water for 3 min, followed by a linear gradient from 0 to 50% acetonitrile over 15 min with UV detection at 205 nm.

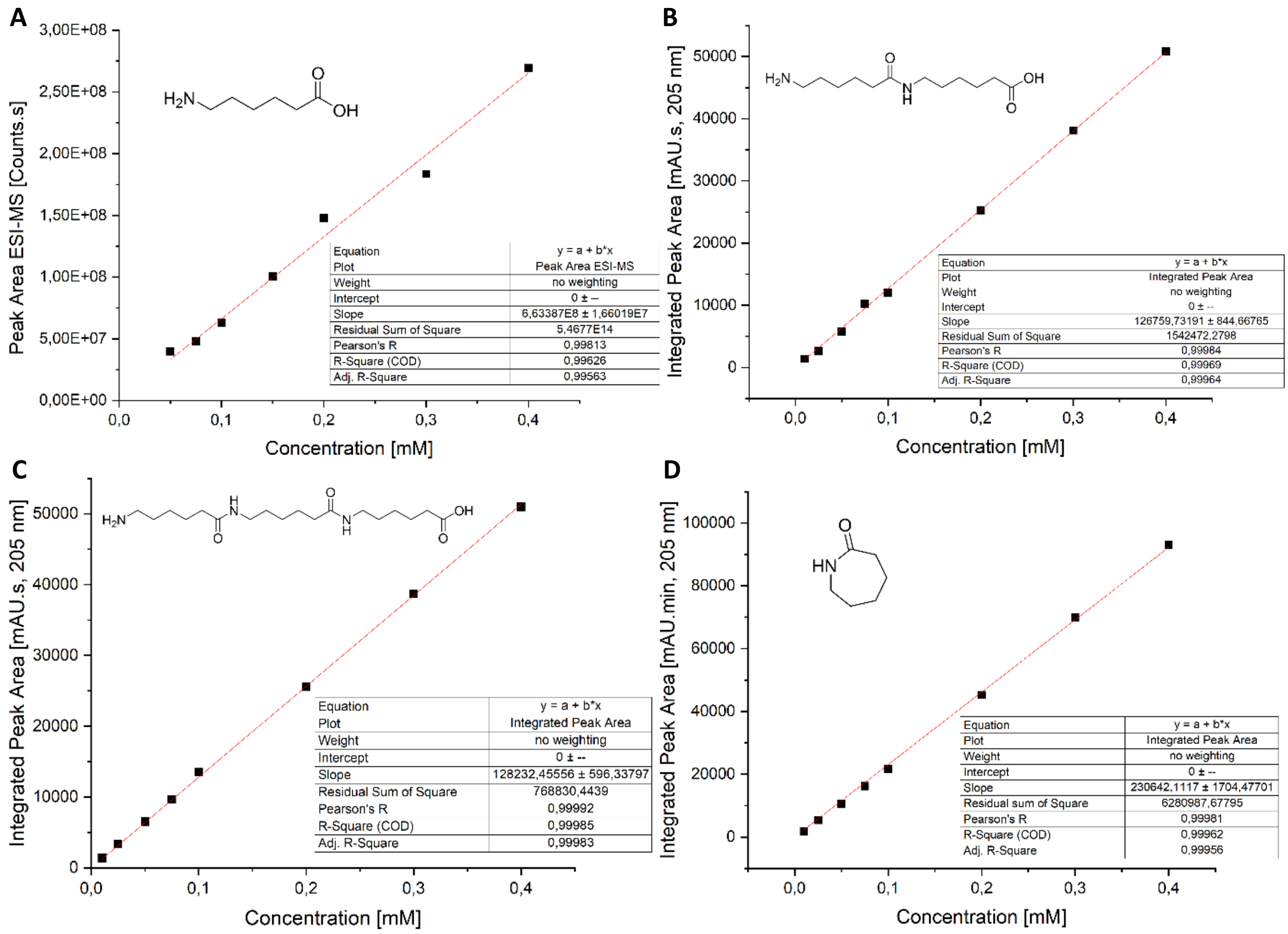

Figure S7. Calibration curves for quantification of PA6 film degradation products by RP-HPLC: (**A**) 6-aminohexanoic acid (6-AHA), (**B**) 6-AHA dimer, (**C**) 6-AHA trimer, and (**D**) *ε*-caprolactam.

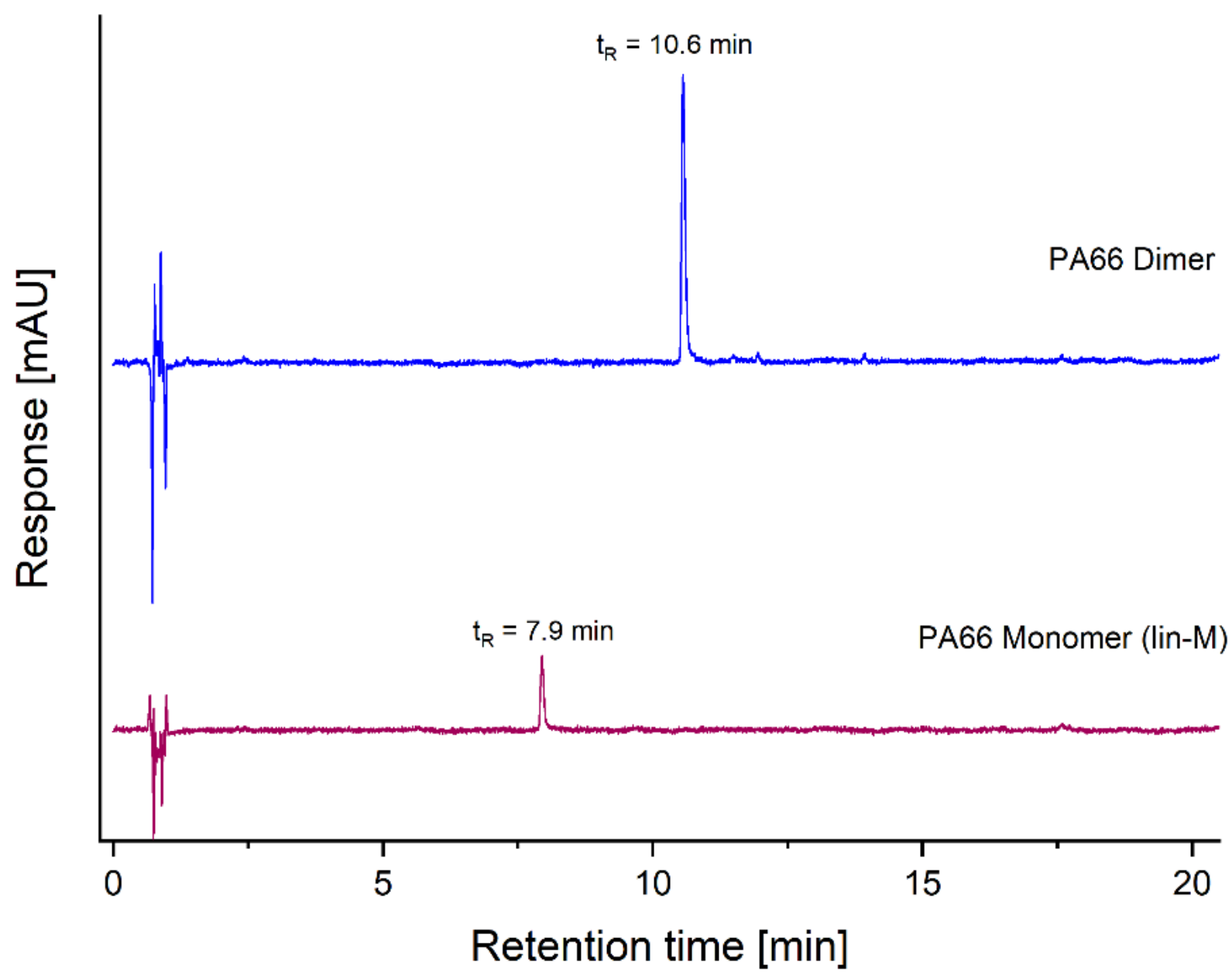

Figure S8. RP-HPLC method for the detection of PA66 granule degradation products. Chromatograms of linear PA66 repeat-unit product (lin-M) (t_R = 7.9 min) and PA66 dimer (t_R = 10.6 min), each at 0.1 mM. Chromatograms were recorded using an isocratic hold at 100% water for 3 min, followed by a linear gradient from 0 to 50% acetonitrile over 15 min with UV detection at 205 nm.

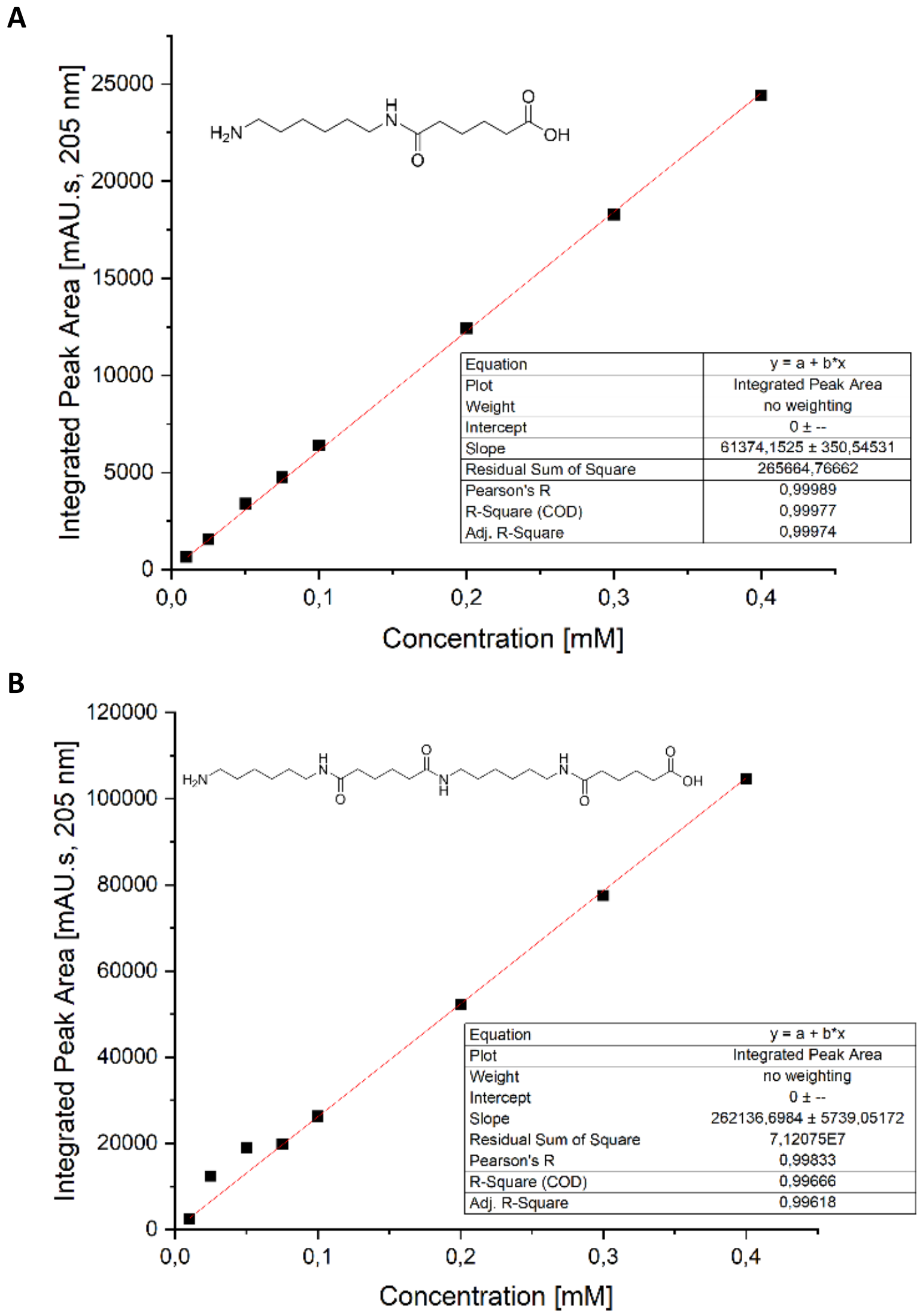

Figure S9. Calibration curves for quantification of PA66 granule degradation products by RP-HPLC: (**A**) linear PA66 repeat-unit product (lin-M) and (**B**) PA66 dimer. For quantification of the cyclic monomeric species (cy-Mono), the calibration curve of lin-M was used as a surrogate standard, whereas the cyclic dimer (cy-Dimer) and the diacid-terminated product (M–AA) were quantified using the calibration curve of the PA66 dimer.

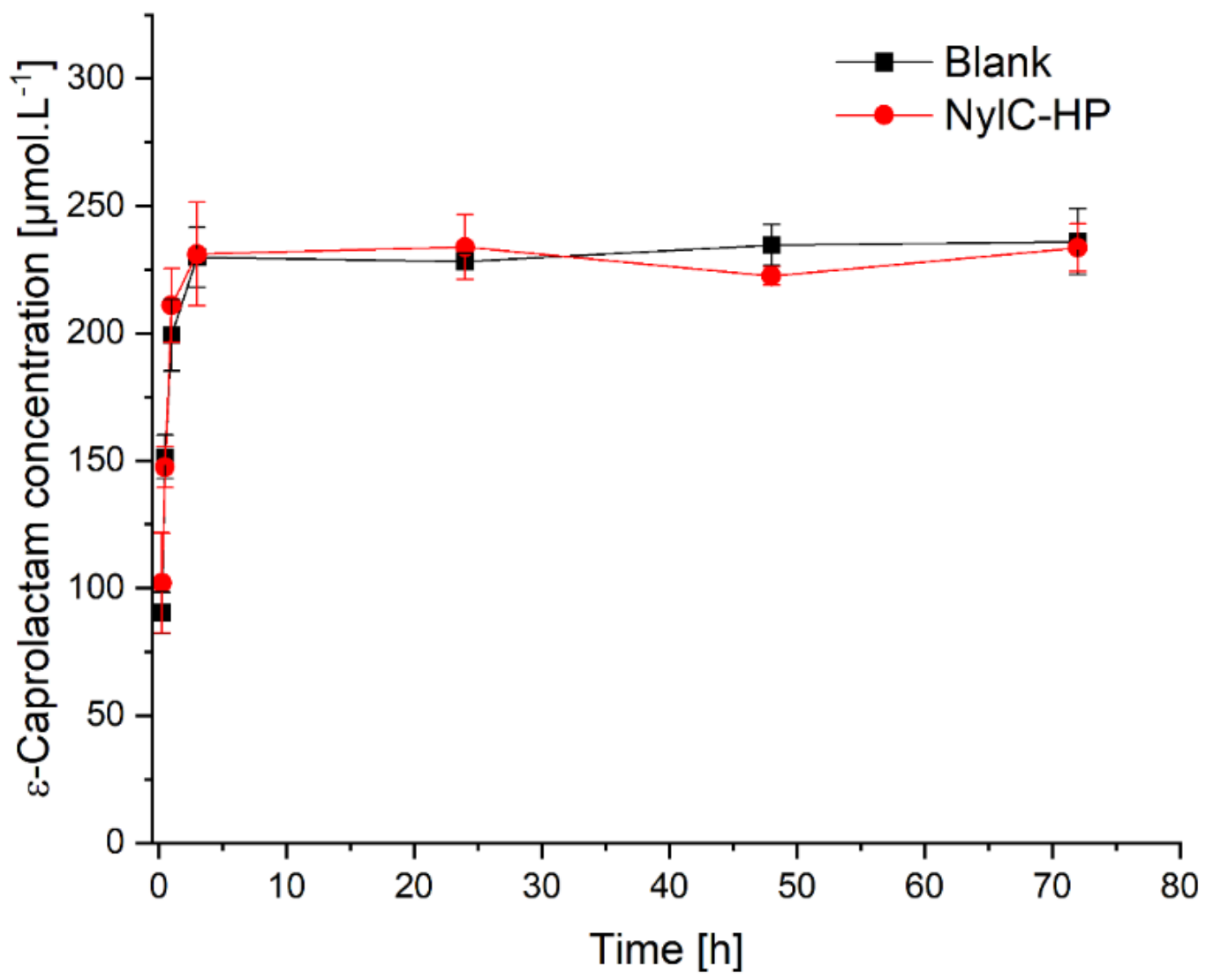

Figure S10. Time-dependent formation of *ε*-caprolactam during incubation of PA6 film in the absence of enzyme (blank, black) and in the presence of NylC-HP (red) at 70°C. Error bars represent the standard deviation of replicate measurements.

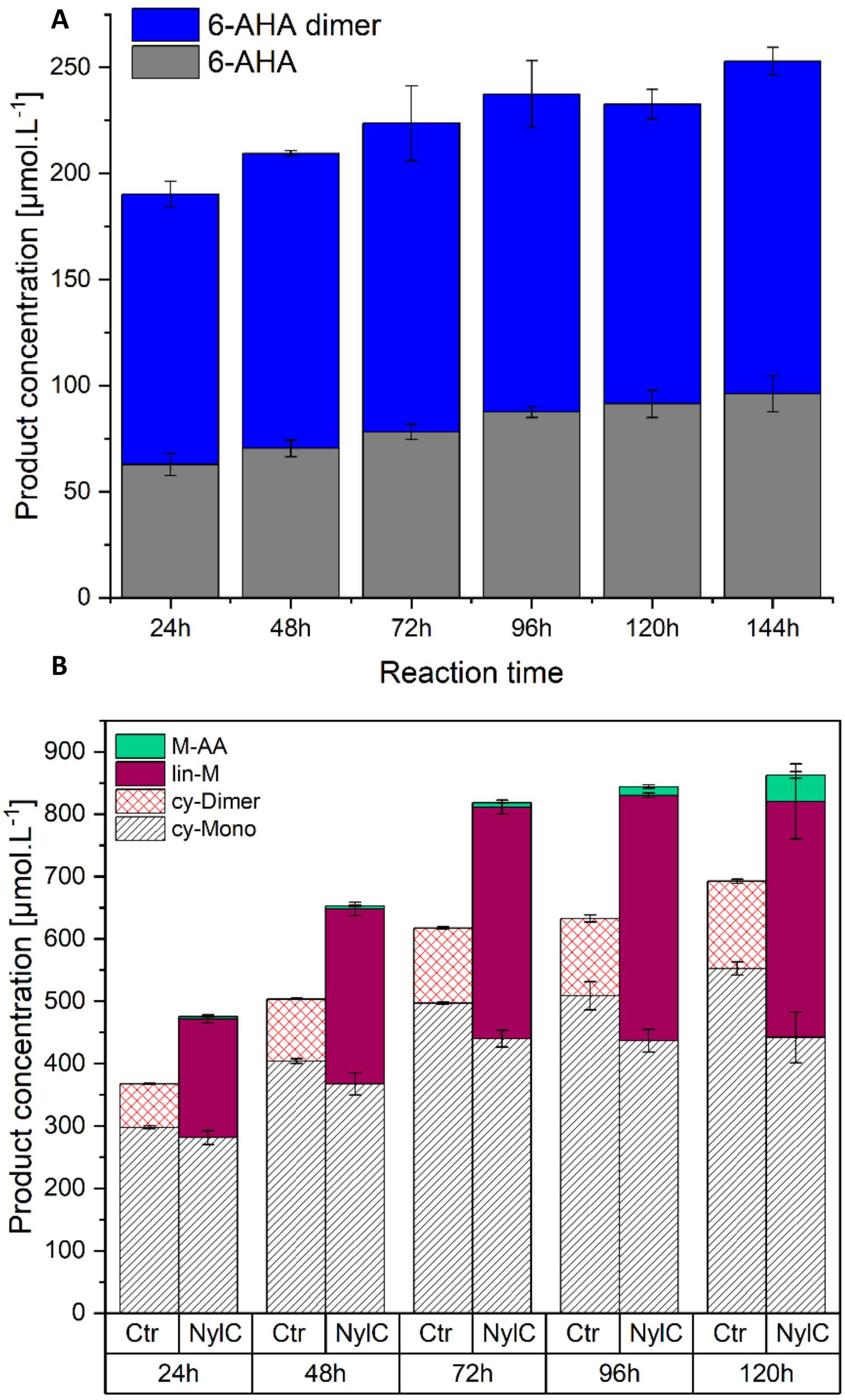

Figure S11. Time-dependent formation of hydrolysis products from bulk nylon substrates using NylC-HP. (**A**) Product distribution from PA6 film incubated with 1.1 µM enzyme. (**B**) Product distribution from PA66 granule incubated with 1.65 µM enzyme. Reactions were performed in 100 mM potassium phosphate buffer (pH 7.5, 150 mM NaCl) at 70°C. All assays were performed in triplicate (*n* = 3); error bars represent the standard deviation of replicate measurements.

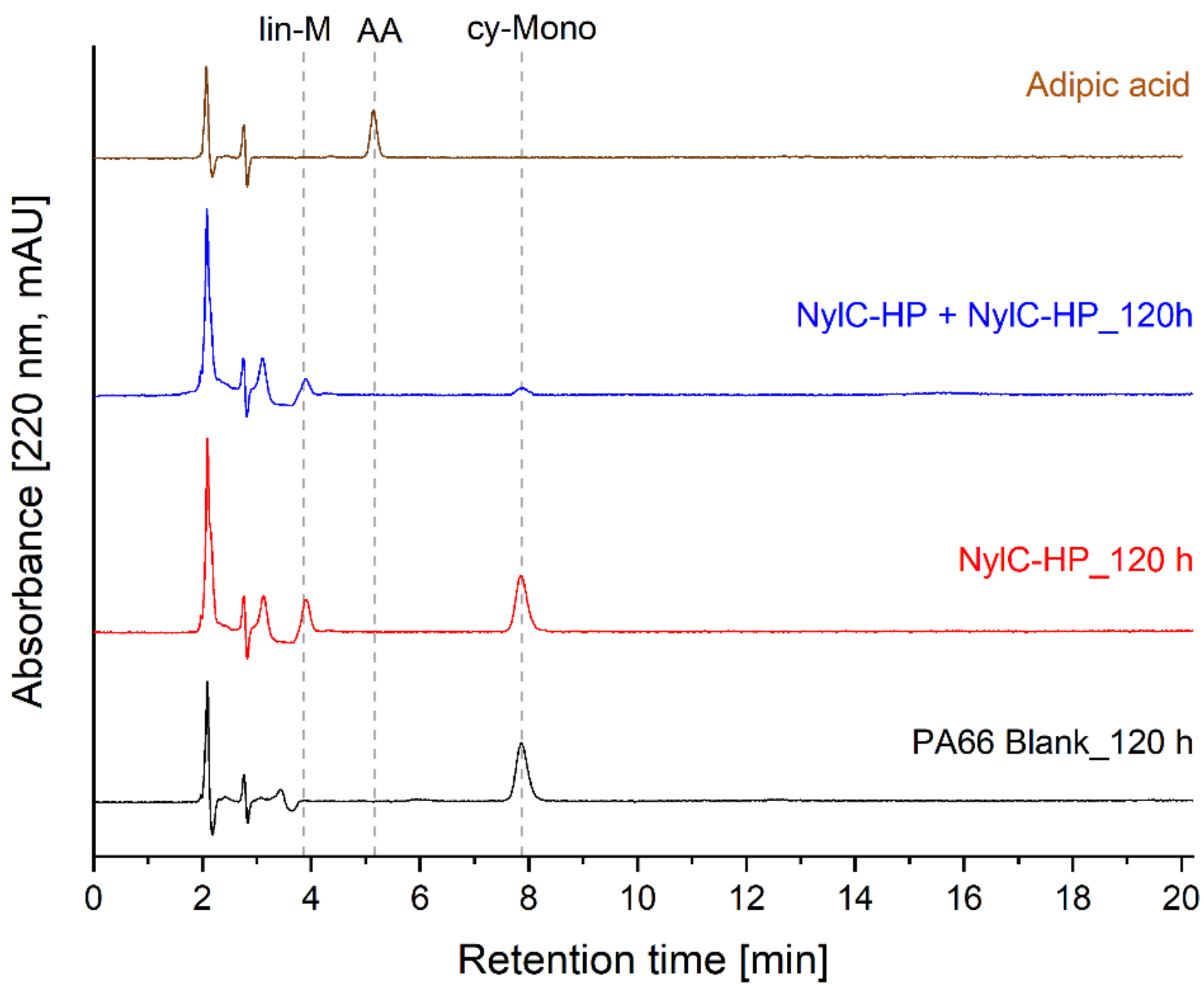

Figure S12. RP-HPLC chromatograms of PA66 granule degradation samples after 120 h. Shown are PA66 blank (no enzyme), the NylC-HP-treated sample, sequential treatment (re-addition of NylC-HP), and adipic acid (AA) standard (1 mM). Peaks corresponding to the linear PA66 repeat-unit product (lin-M), adipic acid (AA), and the cyclic monomeric species (cy-Mono) are indicated. Chromatograms were recorded under isocratic conditions using 10% acetonitrile over 20 min with UV detection at 220 nm.

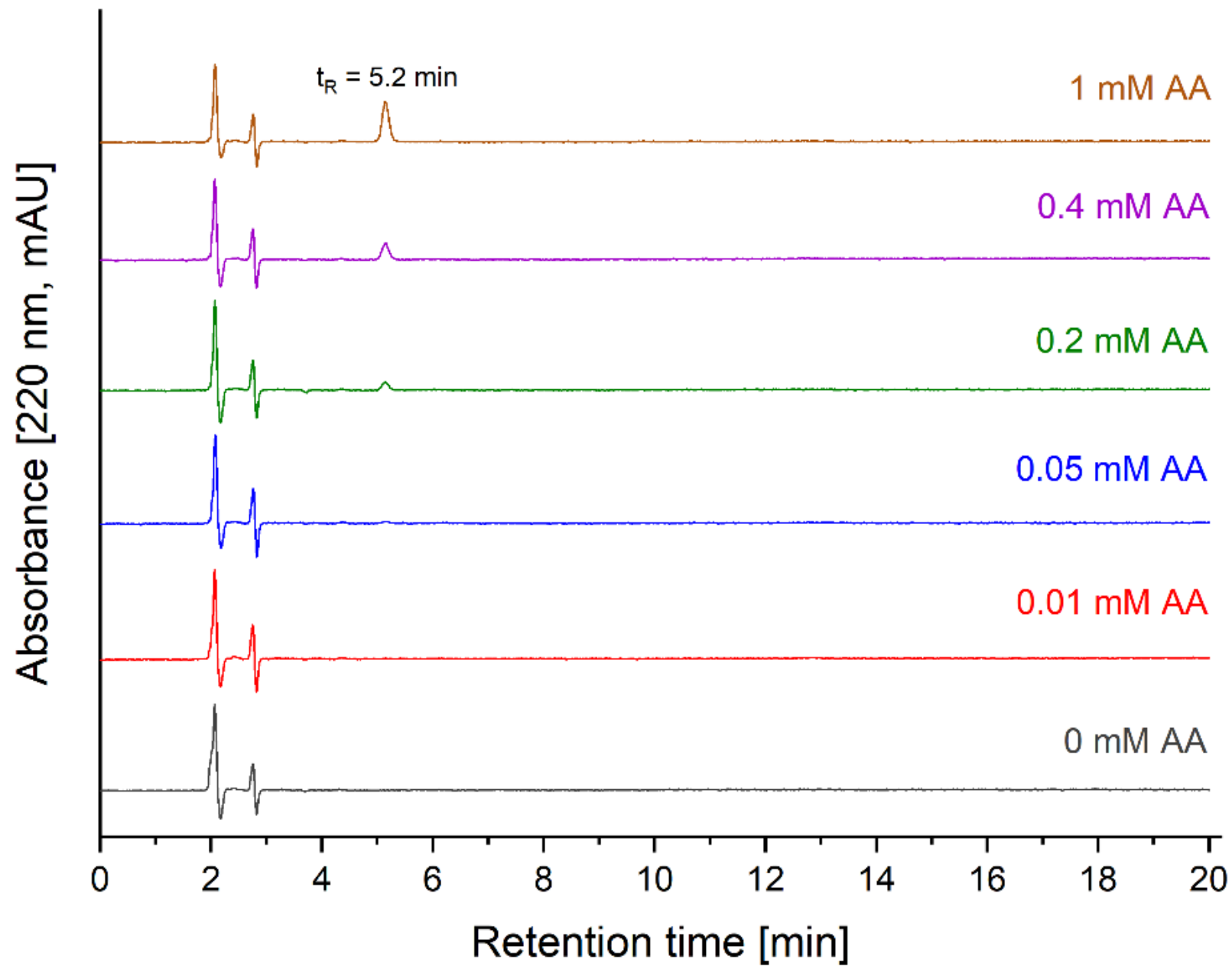

Figure S13. RP-HPLC chromatograms of AA standards at concentrations ranging from 0 to 1 mM. Peak intensity increases with concentration, with AA eluting at $t_R \approx 5.2$ min. Chromatograms were recorded with UV detection at 220 nm.

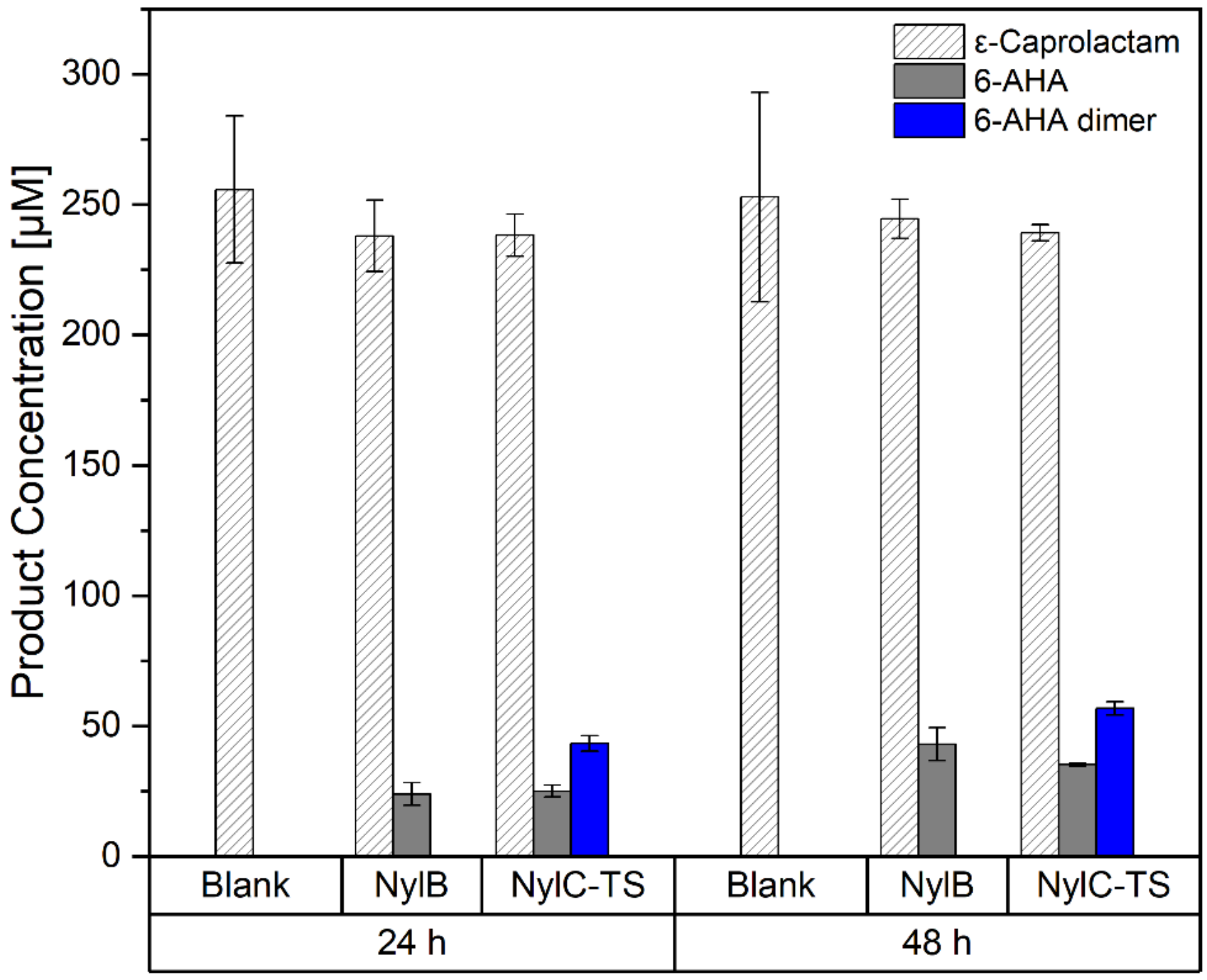

Figure S14. Time-dependent formation of soluble PA6-derived products from PA6 film in the absence of enzyme (Blank) and in the presence of NylB or $NylC_{p2}$-TS at 40°C. All assays were performed in triplicate (*n* = 3); error bars represent the standard deviation of replicate measurements.

References

[1] Wypych, A.; Bäse, N.; Feuerriegel, G.; Wonneberger, C. F. E.; Awan, M. R. A.; Graack, G.; Menzel, J.; Chibani, C. M.; Dewi, S.; Thies, S.*; et al.* Functional mining in marine ecosystems uncovers unexpected diversity of globally distributed nylon degrading enzymes. *Manuscript submitted for publication* **2026**.

[2] Puetz, H.; Illig, A.-M.; Vorobii, M.; Janknecht, C.; Contreras, F.; Flemig, F.; Schwaneberg, U. KnowVolution of an Efficient Polyamidase through Molecular Dynamics Simulations of Incrementally Docked Oligomeric Substrates. *ChemSusChem* **2025**, *18* (15), e202500257. DOI: 10.1002/cssc.202500257.

[3] Yasuhira, K.; Tanaka, Y.; Shibata, H.; Kawashima, Y.; Ohara, A.; Kato, D.-I.; Takeo, M.; Negoro, S. 6-Aminohexanoate oligomer hydrolases from the alkalophilic bacteria Agromyces sp. strain KY5R and Kocuria sp. strain KY2. *Appl. Environ. Microbiol.* **2007**, *73* (21), 7099–7102. DOI: 10.1128/AEM.00777-07.

[4] Negoro, S.; Kato, K.; Fujiyama, K.; Okada, H. The nylon oligomer biodegradation system of Flavobacterium and Pseudomonas. *Biodegradation* **1994**, *5*, 185–194.

[5] Bradford, M. M. A Rapid and Sensitive Method for the Quantitation of Microgram Quantities of Protein Utilizing the Principle of Protein-Dye Binding. *Anal. Biochem.* **1976**, *72*, 248–254.

[6] Kinoshita, S.; Negoro, S.; Muramatsu, M.; Bisaria, V. S.; Sawada, S.; Okada, H. 6-Aminohexanoic acid cyclic dimer hydrolase. A new cyclic amide hydrolase produced by Achromobacter guttatus KI74. *Eur. J. Biochem.* **1977**, *80* (2), 489–495. DOI: 10.1111/j.1432-1033.1977.tb11904.x.

[7] Negoro, S.; Shibata, N.; Tanaka, Y.; Yasuhira, K.; Shibata, H.; Hashimoto, H.; Lee, Y.-H.; Oshima, S.; Santa, R.; Mochiji, K.*; et al.* Three-dimensional structure of nylon hydrolase and mechanism of nylon-6 hydrolysis. *J. Biol. Chem.* **2012**, *287* (7), 5079–5090. DOI: 10.1074/jbc.M111.321992.

[8] Han, W.; Govignon, Q.; Cantarel, A.; Schmidt, F. Efficient polymerization and crystallization kinetics coupling of polyamide 6 synthesis for liquid composite molding process modeling. *Polym. Eng. Sci.* **2022**, *62* (4), 999–1012. DOI: 10.1002/pen.25901.

[9] Blaine, R. L. Polymer Heats of Fusion. *TA Instruments* **2002**, New Castle.
